# Machine Learning in Causal Effect Estimation and Causal Evaluation of ML‐Enabled Interventions in Nursing‐Related Research: A Scoping Review

**DOI:** 10.1111/nhs.70431

**Published:** 2026-10-07

**Authors:** Dimitrios Kosmidis, Georgios Vlachopoulos, Abdulqadir J. Nashwan, Rafael Lima Rodrigues de Carvalho, Breno Santos Araujo, Dimitra Armeni, Eleni Sertaridou, Sotiria Koutsouki

**Affiliations:** ^1^ Department of Nursing Democritus University of Thrace Alexandroupolis Greece; ^2^ Nursing & Midwifery Research Department Hamad Medical Corporation Doha Qatar; ^3^ Escola de Enfermagem Universidade Federal da Bahia Salvador Bahia Brazil; ^4^ Department of Genomics Erasmus University Rotterdam Rotterdam the Netherlands; ^5^ Medical Department Democritus University of Thrace Alexandroupolis Greece; ^6^ Intensive Care Unit University Hospital of Alexandroupolis Greece; ^7^ Intensive Care Unit General Hospital of Kavala Kavala Greece

**Keywords:** causal effect estimation, causal inference, causal machine learning, intervention evaluation, machine learning, nursing research, scoping review

## Abstract

Machine learning (ML) informs nursing practice, but prediction cannot establish intervention or workflow effects. Aim To map ML use within causal effect estimation and causal evaluations of ML‐enabled interventions in nursing and midwifery. Eight electronic sources were searched for reports published January 2015–June 2026, followed by backward citation searching. Eligibility required a causal estimand, corresponding identification strategy, substantive ML role, and nursing or midwifery relevance. Two reviewers independently screened records and full texts. Fifteen studies (17 reports) were included. Five addressed acute deterioration or sepsis; others covered care prioritization, falls, post‐discharge care, and nurse‐ or midwife‐led services. Five used ML within causal effect estimation to examine longitudinal and spillover effects, treatment‐effect heterogeneity, and targeting strategies. Ten evaluated ML‐enabled interventions or workflows; reported favorable effects on mortality, care escalation, message‐review time, and nursing processes, while several controlled estimates were null or imprecise. Evidence remains small and methodologically uneven. Future studies should align estimands, identification strategies, analytic methods, and workflows and evaluate model‐plus‐workflow effects on nursing processes and patient outcomes.

## Key Points

1. Across the included studies, ML occupied two distinct roles: it was used within causal effect estimation or embedded in an intervention or workflow whose causal effect was evaluated.
2. Nursing‐ and midwifery‐related research of this type remains limited, with only 15 studies identified and mixed findings, including some favorable effects but several null or imprecise controlled estimates.
3. Future studies should align causal estimands, identification strategies, analytic methods, and workflows, and evaluate model‐plus‐workflow effects on nursing processes and patient outcomes.

## 1 Introduction

Machine learning (ML) is now used across nursing‐ and midwifery‐related research for risk prediction, patient monitoring, documentation, clinical decision support, workflow management, and education. Of note, Choi et al. (2025) mapped the algorithms, model‐evaluation practices, and application areas represented in nursing research. Kim and Kim (2025) examined methodological characteristics and reporting quality and assessed progress toward clinical implementation in nursing‐affiliated ML research. Kim et al. (2025) reviewed machine‐learning‐based prediction models using nursing data in adult intensive care units, focusing on model characteristics and the types of nursing data used. Importantly, in a review of deterioration‐prediction systems, Peelen et al. (2021) showed that model development and predictive validation are only part of the evidence needed once an algorithm is introduced into clinical care, where workflow integration and effects on practice and outcomes become central.

Prediction and causal effect estimation answer different questions. A predictive model estimates who is likely to experience an outcome under the patterns of care represented in the available data. It does not, by itself, determine what would happen if clinicians used the model, changed a workflow, allocated an intervention, or selected one care strategy rather than another. Such questions require comparison of well‐defined alternative intervention or exposure scenarios, as distinguished from outcome prediction by Fernández‐Loría and Provost (2022) and Feuerriegel et al. (2024). In essence, this distinction between prediction and causal effect estimation is particularly important in nursing and midwifery because ML outputs may influence prioritization, monitoring, escalation, communication, care coordination, education, or service delivery. The practical value of a model therefore depends not only on predictive performance but also on the consequences of acting on its output within a specific care context.

Causal inference concerns drawing conclusions about the effects of well‐defined interventions or exposures by comparing outcomes under alternative scenarios (Dahabreh and Bibbins‐Domingo 2024; Hernán and Robins 2020). In this review, causal effect estimation required an intervention‐ or exposure‐oriented question, a defined causal estimand, and a corresponding identification strategy linking that estimand to an observed‐data contrast under explicit assumptions. A causal estimand specifies the intervention or exposure, comparator, outcome, target population, time horizon, and effect measure. It may concern an average treatment effect across a target population or a conditional average treatment effect within groups defined by baseline characteristics. The identification strategy comprises the design features and assumptions that allow the causal estimand to be expressed as an observed‐data quantity. The estimator is the statistical procedure used to estimate that quantity (Dahabreh and Bibbins‐Domingo 2024; Dang et al. 2023). For example, consider a hypothetical study examining whether an ML‐enabled delirium‐risk alert linked to a nurse‐led prevention bundle reduces incident delirium among older hospitalized adults. The causal estimand would be the difference in the risk of developing delirium during hospitalization (outcome) under implementation of the ML‐enabled alert and nurse‐led prevention bundle (intervention) versus usual care without the alert (comparator) among adults aged 65 years or older who are free of delirium at admission (target population) from admission to hospital discharge (time horizon), expressed as an absolute risk difference (effect measure).

In a cluster‐randomized trial, random assignment of hospital wards to the intervention or control condition would constitute the principal identification strategy (design feature), under assumptions such as consistency, absence of interference between wards, and comparable outcome ascertainment across groups (identifying assumptions). The effect could then be estimated using a regression model comparing delirium risk between the randomized groups while accounting for clustering at the ward level (estimator). This example illustrates how the causal estimand defines the effect of interest, the identification strategy establishes how that effect can be identified from the observed data, and the estimator provides the statistical procedure used to estimate it.

Neither causal terminology nor the use of an advanced statistical method is sufficient, by itself, to justify a causal interpretation. This distinction is central to the frameworks described by Dang et al. (2023), Dahabreh and Bibbins‐Domingo (2024), and Hernán and Robins (2020). Core causal and causal ML terms used in this review are summarized in Box 1.

ML may occupy one of two distinct positions within this framework. First, in Category 1, ML may contribute directly to causal effect estimation by flexibly modeling potential outcomes, treatment‐assignment or censoring mechanisms, or variation in effects across individuals or subgroups. Super Learner may be used to estimate nuisance functions flexibly, while causal forests, meta‐learners, and counterfactual representation‐learning models may support estimation of heterogeneous effects (Feuerriegel et al. 2024; Künzel et al. 2019; Wager and Athey 2018). These methods can reduce reliance on restrictive functional‐form assumptions, but they do not replace a clearly defined estimand, a corresponding identification strategy, and plausible identifying assumptions. ML can strengthen estimation after the causal question and identification strategy have been specified, but it cannot by itself establish identification (Dang et al. 2023; Moccia et al. 2024).

Second, in Category 2, ML may be a substantive component of the intervention, tool, workflow, or targeting strategy whose effect is being evaluated. In this case, the ML model may generate an alert, risk category, ranking, recommendation, or educational response that guides subsequent user actions. The causal effect may then be estimated through a randomized or quasi‐experimental design without ML forming part of the estimator. The relevant intervention is not the algorithm in isolation, but the implemented model‐plus‐workflow package, including how outputs are presented, who receives them, what actions they trigger, and how those actions interact with existing care processes. This interpretation is consistent with reporting and evaluation guidance for artificial intelligence (AI) interventions. Cruz Rivera et al. (2020) and Liu et al. (2020) emphasize intended use, users, inputs, outputs, and human–AI interaction in the Standard Protocol Items: Recommendations for Interventional Trials–Artificial Intelligence (SPIRIT‐AI) and Consolidated Standards of Reporting Trials–Artificial Intelligence (CONSORT‐AI) extensions. Vasey et al. (2022) extend this emphasis in the Developmental and Exploratory Clinical Investigations of DEcision support systems driven by Artificial Intelligence (DECIDE‐AI) guideline to early live clinical evaluation, workflow integration, human factors, and implementation context.

Thus, studies in which ML is used within the causal effect‐estimation procedure primarily ask about the magnitude or heterogeneity of an intervention effect, whereas studies evaluating an ML‐enabled system ask about the effect of implementing a model within a defined workflow. Given the conceptual and methodological heterogeneity of this emerging literature, a scoping review was undertaken to map the available evidence in both cases described above. The review aimed to map the applications, causal estimands, identification strategies, ML roles, nursing or midwifery relevance, and recurring methodological and reporting gaps in the literature.

BOX 1 **Core Causal and Causal ML Terms Used in This Review.**

**Table:** 

Term	Definition
Causal estimand	The precise causal quantity a study aims to estimate, defined by the intervention or exposure, comparator, outcome, target population, time horizon, and effect measure (Dahabreh and Bibbins‐Domingo 2024; Hernán and Robins 2020).
Causal inference	The process of drawing conclusions about the effects of well‐defined interventions or exposures by specifying a causal estimand, identifying it under explicit assumptions, and estimating the corresponding observed‐data quantity (Dahabreh and Bibbins‐Domingo 2024; Hernán and Robins 2020).
Estimator	The statistical procedure used to estimate the identified observed‐data quantity from a sample. Different estimators may target the same estimand (Dang et al. 2023).
Causal effect	A contrast between potential outcomes under two or more well‐defined alternative intervention or exposure scenarios in a specified target population and time horizon (Hernán and Robins 2020).
Identification strategy	The design features and assumptions that allow a causal estimand to be expressed as a statistical quantity in the observed‐data distribution. It is distinct from the estimator used to calculate that quantity (Dahabreh and Bibbins‐Domingo 2024; Dang et al. 2023).
Potential outcome (counterfactual outcome)	The outcome that would be observed for a person, cluster, or population under a specified intervention or exposure scenario. For a given unit and time, only the potential outcome corresponding to the scenario actually experienced is observed (Hernán and Robins 2020).
Average treatment effect (ATE)	The mean difference between the potential outcomes under the intervention and comparator across the target population (Hernán and Robins 2020).
Conditional average treatment effect (CATE)	The mean difference between potential outcomes under the intervention and comparator within a subgroup defined by baseline characteristics (Feuerriegel et al. 2024; Hoogland et al. 2021).
Individual treatment effect (ITE)	The difference between an individual's potential outcomes under the intervention and comparator. Because both potential outcomes cannot be observed for the same individual, ITE estimates depend on modeling assumptions (Feuerriegel et al. 2024; Hoogland et al. 2021).
Causal machine learning (causal ML)	The use of ML methods within a causal analysis to estimate potential outcomes, treatment‐effect functions, or nuisance functions required by a causal estimator. In this review, the term corresponds to Category 1 and does not encompass every causal evaluation of an ML‐enabled intervention in Category 2 (Feuerriegel et al. 2024).

## 2 Methods

### 2.1 Review Design, Questions, and Population–Concept–Context Framework

This scoping review was conducted in accordance with the Joanna Briggs Institute (JBI) methodology for scoping reviews (Peters et al. 2024) and reported in accordance with the Preferred Reporting Items for Systematic Reviews and Meta‐Analyses extension for Scoping Reviews (PRISMA‐ScR) (Tricco et al. 2018). The completed checklist is provided as Appendix S1, PRISMA‐ScR Checklist. No protocol was prospectively registered or made publicly available.

The primary review question was, “What empirical nursing‐ or midwifery‐related studies have used ML within causal effect estimation or evaluated the causal effect of an ML‐enabled intervention, tool, workflow, or targeting strategy?” A secondary question was, “What are the methodological and contextual characteristics of these studies?”

The review questions and eligibility criteria were structured using the Population–Concept–Context (PCC) framework (Peters et al. 2024). Detailed PCC‐aligned eligibility criteria are presented below. Because the Population element was unrestricted, no population search block was used.

### 2.2 Eligibility Criteria

Eligibility was determined at the study level. At least one full report had to independently satisfy all applicable eligibility criteria described below and provide sufficient information to assess the causal estimand, identification strategy, substantive ML role, and nursing or midwifery relevance against the predefined criteria. Reports describing the same underlying study were linked. Once study eligibility had been established, an additional companion report was retained when it met the report‐level criteria under Types of evidence sources and contributed distinct information on the causal estimand, identification strategy, estimator, ML role, or findings relevant to the review questions. Companion reports were not counted as separate studies. The criteria informed title and abstract screening and were applied definitively during full‐text assessment.

#### 2.2.1 Population

The population was unrestricted. No population‐specific eligibility restriction was applied.

#### 2.2.2 Concept

The Concept element was causal effect estimation, with ML occupying one of two qualifying roles, either within the causal effect estimation procedure or as a functional component of an intervention, tool, workflow, or targeting strategy whose causal effect was evaluated.

Causal estimand and identification strategy criterion. Studies had to specify a causal estimand and a corresponding identification strategy, consistent with frameworks that distinguish causal question specification, identification, and statistical estimation (Dahabreh and Bibbins‐Domingo 2024; Dang et al. 2023). Use of the term estimand was not required, but the intervention or exposure, comparator, outcome, target population, relevant time point or follow‐up horizon, and effect contrast or measure had to be clear (Dahabreh et al. 2016; Hernán and Robins 2020). Identification referred to the design features and assumptions linking the causal estimand to an observed‐data contrast before selection of an estimator (Dahabreh and Bibbins‐Domingo 2024; Dang et al. 2023).

Eligible approaches included randomization, including cluster and stepped‐wedge designs, and explicit non‐randomized designs or strategies, such as target‐trial emulation, regression discontinuity, difference‐in‐differences, interrupted, and Bayesian structural time series with an explicit counterfactual, instrumental variables, and synthetic controls. G‐methods for time‐varying confounding were also eligible (Hernán and Robins 2020).

For non‐randomized studies, the assignment mechanism, comparison structure, and relevant design or confounding‐control strategy had to be described sufficiently to identify the assumptions on which the causal contrast depended. Formal labels or a complete enumeration of those assumptions were not required. Matching, weighting, or regression adjustment could contribute to an exchangeability‐based identification strategy but were insufficient when presented without a defined causal estimand and corresponding assumptions. Where a study combined such adjustment with a design‐based comparison, eligibility rested on the design‐based component. Causal terminology and uncontrolled before‐and‐after comparisons were likewise insufficient. Causal discovery or graph‐learning studies without a defined intervention or exposure contrast and causal effect estimand were excluded because they did not address the causal quantity required by this review (Feuerriegel et al. 2024).

Qualifying ML role. ML had to have a substantive and clearly described role in one of two qualifying categories. For this review, generic AI labels, entirely rule‐based systems, and proprietary or hybrid systems without a described learned component were insufficient. The report had to identify at least one learned component and explain its substantive function in the study.

In Category 1, ML had to form part of the primary causal effect estimation procedure by estimating potential outcomes, treatment‐effect functions, or nuisance functions required by the causal estimator. Eligible approaches included causal forests, meta‐learners, and data‐adaptive nuisance estimation within targeted, orthogonal, doubly robust, or related estimators (Feuerriegel et al. 2024; Moccia et al. 2024). Prognostic outcome prediction or risk scoring alone did not qualify because these approaches do not estimate contrasts between potential outcomes under well‐defined alternative interventions or exposures (Feuerriegel et al. 2024).

In Category 2, the study had to evaluate the causal effect of implementing or using an ML‐enabled intervention, tool, workflow, or targeting strategy. The ML output had to guide or trigger allocation, prioritization, escalation, recommendation, decision‐making, or another clinical, educational, or operational action. The causal contrast could concern the total effect of a multicomponent model‐plus‐workflow package, provided that ML was a functional component contributing to the downstream action. The identification strategy and estimator did not themselves need to use ML because the ML component was located in the intervention or workflow rather than necessarily in the effect‐estimation procedure. This operationalization was informed by the emphasis of CONSORT‐AI and DECIDE‐AI on intended use, users, outputs, human–AI interaction, workflow integration, and downstream actions (Liu et al. 2020; Vasey et al. 2022).

ML used only for standalone prediction, cohort construction, recruitment, eligibility screening, preprocessing, ancillary adjustment outside the primary causal estimator, or supplementary sensitivity analysis did not qualify. ML‐based eligibility or enrichment qualified only when the causal contrast evaluated the ML‐guided targeting strategy itself (Fernández‐Loría and Provost 2022). An ML‐derived exposure, outcome, phenotype, or other measurement was not an independent inclusion pathway, although it could be charted when the study otherwise qualified under Category 1 or 2 (Feuerriegel et al. 2024).

#### 2.2.3 Context

The Context element comprised nursing‐ and midwifery‐related care, workflows, decision support, documentation, monitoring, care organization, education, and training.

Nursing or midwifery relevance criterion. Studies had to satisfy at least one of four non‐mutually exclusive pathways. Intervention‐role relevance required nurses or midwives to lead, coordinate, or substantively implement the intervention or service model. End‐user relevance required an ML‐enabled output to be delivered to nurses or midwives who were expected to interpret or act on it within an actual workflow. Registered nurses in advanced practice roles were treated as nursing end users, provided that the nursing role in receiving or acting on the output was documented in the primary report.

Data‐and‐question relevance required both nursing or midwifery data as a primary analytic source and a causal question directly concerning nursing or midwifery care, assessment, monitoring, documentation, or decision‐making. Educational relevance required an ML‐enabled application in nursing or midwifery education or training with discipline‐specific outcomes. In multiprofessional educational samples, nursing or midwifery findings had to be distinguishable. The inclusion of nurses, midwives, or nursing or midwifery students in the study population, the setting, publication venue, isolated nursing variables, or incidental execution of medically directed care did not establish relevance. Detailed operational rules are provided in Box S1.

#### 2.2.4 Types of Evidence Sources

Eligible reports were English‐language full‐text empirical journal articles, full conference papers, dissertations, or theses published from 1 January 2015 to 11 June 2026. Abstracts without an available full report, reports based exclusively on simulated or synthetic data, preprints, protocols, reviews, commentaries, editorials, conceptual or ethical analyses, guidelines, and other non‐empirical publications were excluded.

### 2.3 Information Sources and Search Strategy

Eight electronic sources were searched. PubMed, Scopus, Embase, CINAHL, and Web of Science Core Collection were treated as bibliographic databases. IEEE Xplore and the ACM Digital Library were searched as specialized computer science sources. ProQuest Dissertations & Theses Global was included as a gray literature source to broaden coverage beyond journal and conference publications and to identify eligible dissertations and theses.

Initial electronic searches were completed on 28 September 2025. All eight sources were subsequently rerun using expanded strategies, and the final electronic searches were completed on 11 June 2026. The expanded search results formed the final electronic‐search set used for screening and PRISMA reporting. Because the population was unrestricted, the search operationalized the Concept and Context elements of the PCC framework. The strategies combined terms for causal inference, causal effects, relevant causal designs and estimators, ML, AI, predictive models, clinical decision‐support systems, nursing, and midwifery. Controlled vocabulary was used where available, and each strategy was adapted to the indexing and technical requirements of the source. No language filter was applied at the search stage. Searches covered records published from 1 January 2015 to 11 June 2026. Records not reported in English were excluded at title and abstract screening, and the availability of an English‐language full report was confirmed at full‐text assessment. Complete strategies, search fields, limits, dates, and retrieval counts are provided in Table S1.

Given the heterogeneous and inconsistently indexed terminology for causal methods, ML systems, and nursing workflows, backward citation searching was undertaken. The reference lists of all reports included through electronic searches were screened. Four prior reviews were also selected because they covered complementary areas directly relevant to the review scope: ML algorithms, model‐evaluation methods, and application areas in nursing research (Choi et al. 2025), methodological characteristics, reporting quality, and clinical implementation of ML in nursing‐affiliated research (Kim and Kim 2025), ML‐based prediction modeling using nursing data in adult intensive care units (Kim et al. 2025), and predictive algorithms for clinical deterioration on adult general wards, including implementation effects, facilitators, and barriers (Peelen et al. 2021). Their reference lists were screened to identify potentially eligible studies that might not have been retrieved through electronic searches.

### 2.4 Search Management and Study Selection

Records retrieved through the electronic searches were imported into Zotero, version 9.0.5, and deduplicated. Records identified through backward citation searching were managed separately, deduplicated within and across citation sources, and checked against the electronic‐search set. Retracted publications and records published before 1 January 2015 were removed from the citation‐search pathway before screening. The same eligibility criteria were applied to both search pathways. No automated screening or prioritization tools were used.

Before screening the expanded search set, two reviewers independently piloted the eligibility definitions and decision rules on 100 records. The pilot clarified the distinction between causal terminology and an explicit causal contrast, substantive and ancillary ML use, and nurse‐led, nurse‐facing, and incidental nursing or midwifery involvement. The reviewers then independently screened all titles and abstracts and assessed potentially eligible full‐text reports. After finalization of the revised criteria, both reviewers independently reassessed all retrieved full texts from both search pathways. Disagreements were resolved through discussion and consensus, and no formal inter‐reviewer agreement statistic was calculated.

The criteria were applied sequentially as report type, causal estimand and identification strategy, qualifying ML role, and nursing or midwifery relevance. Once a definitive exclusion reason was identified, later criteria were not routinely assessed. If an additional exclusion reason had already been identified during full‐text assessment, it was also recorded. Accordingly, full‐text exclusion domains were non‐mutually exclusive.

### 2.5 Data Charting

Data were charted using a standardized form that was piloted on a subset of included reports and refined before full charting. One reviewer extracted the data, and a second checked all items against the full text, with discrepancies resolved by consensus. Charting covered study and report characteristics, companion‐report linkage, setting and population, intervention or exposure and comparator, outcomes and time horizon, causal estimand and identification strategy, assumptions, design and estimator, ML role, nursing or midwifery relevance, intended users, main findings, and study‐reported limitations. For Category 2 studies, ML output, triggered or guided action, workflow integration, human–AI interaction, implementation context, and units of assignment and analysis were also recorded.

### 2.6 Critical Appraisal

No formal risk‐of‐bias or methodological quality appraisal was undertaken because the review aimed to map the evidence rather than assess certainty of effects, consistent with JBI guidance for scoping reviews (Peters et al. 2024). Identification strategies, assumptions, temporal ordering, and study‐reported limitations were summarized descriptively without quality ratings or use as grounds for exclusion.

### 2.7 Data Synthesis

Evidence was summarized descriptively at the study level, with companion reports combined for synthesis. Category 1 method families were organized by principal causal target and estimator family, and Category 2 families by principal identification design. Cross‐study patterns in causal specification, identification, ML role, nursing or midwifery relevance, and findings were synthesized narratively. No meta‐analysis or inferential between‐study comparison was undertaken.

## 3 Results

### 3.1 Study Selection

The electronic searches identified 4918 records, and backward citation searching identified a further 1146 records. After screening and full‐text assessment, 15 unique studies met the eligibility criteria and were represented by 17 included reports, including 15 reports identified through electronic searching and two through backward citation searching (Figure 1).

**FIGURE 1:**
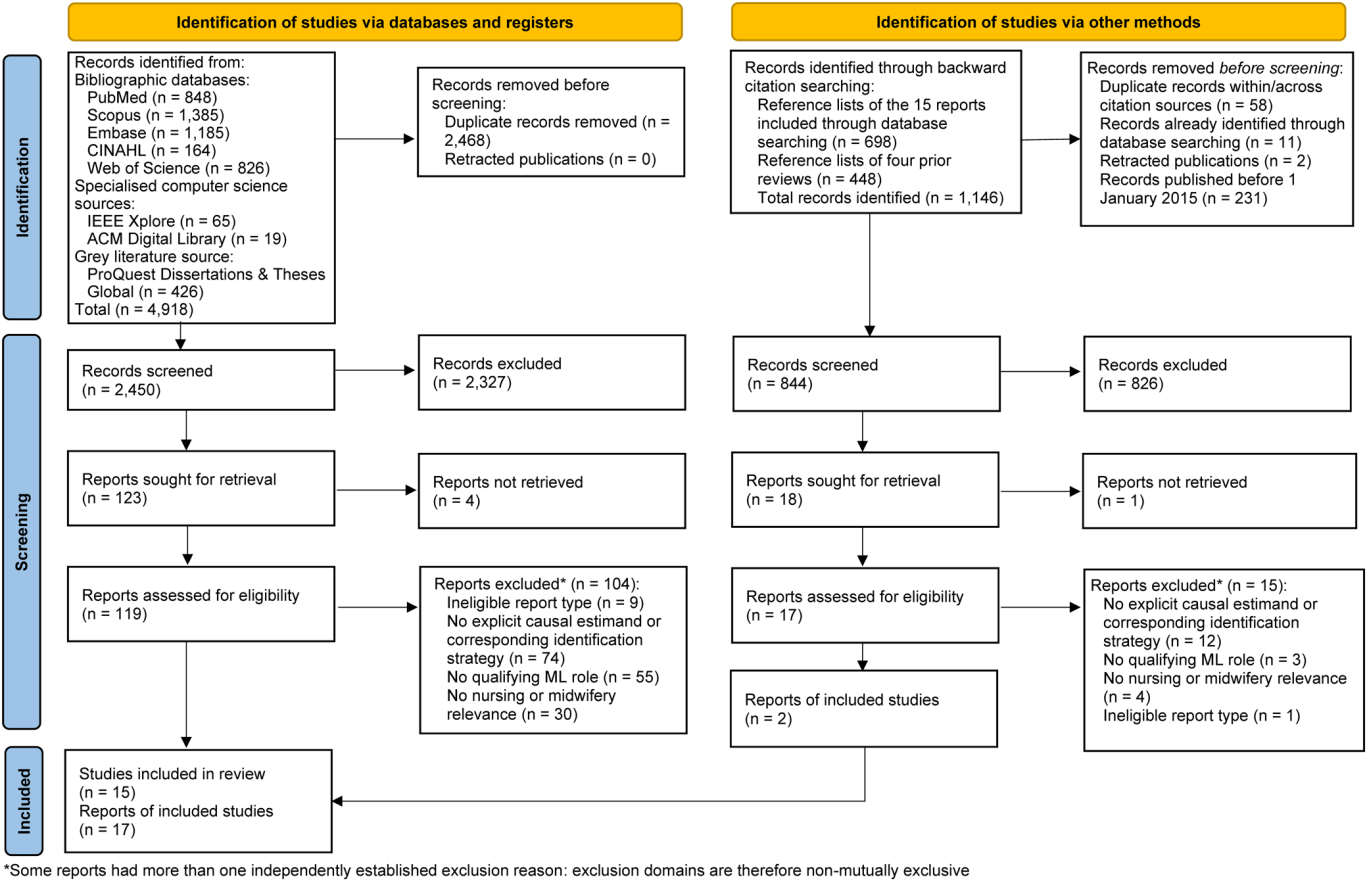
PRISMA 2020 flow diagram.

### 3.2 Characteristics and Analytic Grouping of the Included Studies

Fifteen unique studies were represented by 17 reports published between 2016 and 2026, of which 14 were published from 2020 onward. Five studies, represented by seven reports, used ML within causal effect estimation, while 10 evaluated an ML‐enabled intervention, tool, workflow, or targeting strategy. In Category 1, ML estimated effects over time, accounted for possible spillover between patients, or identified groups with different effects. Methods included longitudinal and interference‐aware estimation, causal forests, meta‐learners, counterfactual neural networks, and recursive partitioning. Category 2 assessed implemented ML systems using randomized or allocated comparisons, regression discontinuity, interrupted or Bayesian structural time series, difference‐in‐differences, and controlled pre‐post designs. Notably, no Category 2 study used ML within the effect‐estimation procedure, and no Category 1 study evaluated an ML‐enabled intervention; the two roles did not co‐occur in any included study. The analytic grouping is presented in Table 1.

**TABLE 1:** Analytic grouping for the descriptive synthesis.

Method family	Defining causal target or identification basis	Guiding analytic question	Included report(s)
Category 1. ML within causal effect estimation			
Longitudinal and interference‐aware effect estimation	Population mean counterfactual outcomes, controlled direct effects, overall effects, and exposure‐reallocation effects under longitudinal treatment strategies	What would outcomes be under alternative program‐availability, enrolment, or allocation strategies over time?	Tran et al. (2016) Miles et al. (2019)
Causal forests and generalized random forests	Average, conditional, and individualized treatment effects, including effect‐based targeting	For whom does the intervention work better, less well, or differently, and could treatment be targeted by expected benefit?	Marafino et al. (2020); Langenberger et al. (2023)
Meta‐learners and counterfactual representation learning	Average and individualized treatment effects estimated with meta‐learners or counterfactual deep‐learning representations	What are the average and individual effects of midwife‐led continuity care on low birth weight?	Moges et al. (2025a, 2025b)
Recursive partitioning and causal‐tree approaches	Data‐adaptive subgroup and heterogeneous treatment‐effect estimation	Which caregiver or patient subgroups experience different effects of the intervention?	Shepherd‐Banigan et al. (2020)
Category 2. Causal evaluation of an ML‐enabled intervention, tool, workflow, or targeting strategy			
Individual‐level randomized or allocated controlled evaluations	Effect of individual assignment to an ML‐enabled intervention versus a concurrent control condition	Does individual assignment to the ML‐enabled intervention affect educational or clinical outcomes?	Shimabukuro et al. (2017) Liu et al. (2026)
Cluster‐randomized and stepped‐wedge evaluations	Effect of unit‐ or site‐level implementation under randomized rollout	Does implementation at cluster level change care processes or patient outcomes?	Barwise et al. (2026) Scott et al. (2025)
Regression‐discontinuity evaluation	Local effect of intervention activation immediately above versus below an algorithmic threshold	What is the local causal effect of activating the ML‐enabled workflow near the decision threshold?	Gallo et al. (2024)
Interrupted time‐series and Bayesian structural time‐series evaluations	Post‐implementation change relative to an expected temporal counterfactual, with or without concurrent control units	Did outcomes change beyond what would have been expected without implementation?	Cho et al. (2021) and Boussina et al. (2024)
Difference‐in‐differences and controlled pre‐post evaluations	Differential change between the ML‐enabled setting and a concurrent or constructed comparison condition	Did outcomes change more in the ML‐enabled setting than in the comparison condition?	Yang et al. (2024), Verma et al. (2024) and Barrett et al. (2025)

*Note:* Categories reflect the substantive role of ML in the reviewed studies. Within Category 1, method families are defined by the principal causal target and estimator family. Within Category 2, method families are defined by the principal identification design. The categories organize the descriptive synthesis and do not represent different levels of methodological rigor. All included studies met the same eligibility criteria for an explicit causal estimand, a corresponding identification strategy, a substantive ML role, and nursing or midwifery relevance. Seventeen reports represented 15 unique studies because Tran et al. (2016) and Miles et al. (2019) were companion reports, as were Moges et al. (2025a, 2025b).

Abbreviation: ML, machine learning.

At the study level, nine studies were conducted in the United States, while one study each was conducted in Canada, China, Ethiopia, Germany, Kenya, and South Korea. Settings comprised hospital‐based acute care (*n* = 8), ambulatory, post‐discharge, or caregiver‐support services (*n* = 3), and maternity services, HIV clinics, nursing homes, and nursing education (*n* = 1 each). Among studies analyzed at the individual level, sample sizes ranged from 40 intensive care nurses to 16 513 patients. Other studies used admissions or hospitalizations, patient messages, facility‐quarters, or nursing‐unit time periods as analytic units, with the largest dataset comprising 1 539 285 hospitalizations. Study characteristics are summarized in Table 2.

**TABLE 2:** Characteristics of the included studies.

Study and country	Study design	Setting, population and sample	Intervention or exposure and comparator	Nursing or midwifery relevance
Category 1. ML within causal effect estimation				
Tran et al. (2016), Miles et al. (2019), Kenya	Observational program evaluation; two companion causal analyses	AMPATH HIV clinics; clinically stable adults eligible for nurse‐based task shifting; Tran included 16 513 eligible patients across 15 clinics, whereas Miles used clinic‐specific analytic cohorts	Nurse‐based task shifting and program availability versus no availability, non‐enrolment, or alternative allocation strategies	Intervention‐role relevance: stable HIV follow‐up tasks were transferred from clinical officers to nurses
Marafino et al. (2020), United States	Retrospective observational study	21 hospitals; adults discharged alive after hospitalization; 1 539 285 hospitalizations	Transitions Program, including 30‐day nursing telephone assessment, versus usual post‐discharge care	Intervention‐role relevance: ongoing nursing assessment was a substantive program component
Langenberger et al. (2023), Germany	Secondary analysis of a multicenter, single‐blind randomized controlled trial	Nine hospitals; adults undergoing hip or knee arthroplasty; 6807 patients	Postoperative electronic patient‐reported outcome monitoring with nurse follow‐up versus the control trial arm	Intervention‐role relevance: a study nurse followed up patients after unfavorable patient‐reported outcome alerts
Moges et al. (2025a, 2025b), Ethiopia	Quasi‐experimental study; two companion reports	Four primary hospitals; pregnant women receiving maternity care; 1166 women	Midwife‐led continuity care versus care provided by different professional groups	Intervention‐role relevance: midwives provided continuity across antenatal, intrapartum, and postnatal care
Shepherd‐Banigan et al. (2020), United States	Post hoc secondary analysis of a randomized controlled trial	One Veterans Affairs medical center; informal caregivers of community‐dwelling older veterans; 241 caregiver‐veteran dyads	HI‐FIVES caregiver skills intervention, including registered‐nurse‐delivered tailored telephone training, versus usual care	Intervention‐role relevance: a registered nurse substantively delivered the tailored telephone‐training component
Category 2. Causal evaluation of an ML‐enabled intervention, tool, workflow, or targeting strategy				
Shimabukuro et al. (2017), United States	Patient‐level randomized controlled trial	Two medical‐surgical intensive care units; adult inpatients; 142 analyzed adults	ML sepsis monitoring with telephone alert to the charge nurse versus the existing rule‐based detector	End‐user relevance: the charge nurse received the alert and initiated the standard sepsis‐evaluation pathway
Liu et al. (2026), China	Exploratory controlled educational study with random‐number‐table allocation	Tertiary‐hospital nursing department; intensive‐care nurses undertaking simulation‐instructor training; 40 nurses	Large‐language‐model pedagogical agent plus blended simulation‐instructor training versus traditional blended training	Educational relevance: intensive‐care nurses were the target learners and outcomes were nursing‐specific
Barwise et al. (2026), United States	Two‐arm stepped‐wedge cluster randomized trial	35 hospital units, including wards, intensive care units, and the emergency department; adults with non‐English language preference and complex care needs; 1421 admissions	ML‐ranked interpreter outreach to bedside nurses versus standard clinician‐initiated interpreter requests	End‐user relevance: bedside nurses received and acted on the targeted secure‐chat prompt
Scott et al. (2025), United States	Prospective stepped‐wedge cluster randomized trial	Four pediatric emergency departments; children with concern for sepsis; 1331 eligible encounters	Visible ML septic‐shock clinical decision support versus silent decision support	End‐user relevance: nurse practitioners were direct intended recipients within the clinical workflow
Gallo et al. (2024), United States	Sharp regression‐discontinuity study	Four general internal medicine units; hospitalized adults near the deterioration‐score threshold; 9938 admitted patients, including 963 in the primary regression‐discontinuity cohort	Epic Deterioration Index alert plus nurse‐physician huddle versus patients immediately below the alert threshold	End‐user relevance: nurses received the alert and co‐conducted the structured deterioration huddle
Cho et al. (2021), South Korea	Non‐randomized controlled interrupted time‐series study	12 medical‐surgical units; adult inpatients; 42 476 admissions contributing to nursing‐unit time‐series analyses	Bayesian‐network fall‐risk tool with alerts and targeted care planning versus STRATIFY‐based assessment	End‐user and intervention‐role relevance: nurses used the predictions and implemented targeted prevention
Boussina et al. (2024), United States	Before‐and‐after quasi‐experimental study	Two emergency departments; adults with sepsis; 6217 patients contributing to monthly outcome series	Nurse‐facing COMPOSER sepsis advisory versus the predicted no‐deployment counterfactual	End‐user relevance: nurses received the advisory and initiated or accelerated escalation
Yang et al. (2024), United States	Pre‐post difference‐in‐differences study	Centralized ambulatory nurse message pools; patient portal messages routed for nurse triage; 396 466 messages	BERT‐based high‐acuity prioritization versus the previous first‐in‐first‐out workflow and contemporaneous low‐scoring messages	End‐user relevance: registered nurses used the output to reorder review and escalate urgent messages
Verma et al. (2024), Canada	Controlled pre‐post study with a contemporaneous difference‐in‐differences comparison	General internal medicine and medical subspecialty units; adult medical inpatients; 13 649 general‐medicine and 8470 comparison admissions	CHARTwatch deterioration alerts and high‐risk pathway versus the pre‐intervention general‐medicine period and contemporaneous subspecialty units without alerts	End‐user relevance: charge and bedside nurses used risk information for monitoring and patient assignment
Barrett et al. (2025), United States	Non‐randomized difference‐in‐differences study	218 nursing homes; long‐stay and short‐stay residents; 94 EHR‐plus‐AI and 124 EHR‐only facilities observed over 11 quarters; facility‐quarter analysis	MatrixCare Clinical Advanced Insights embedded in the EHR versus the same EHR without the AI feature	Data‐and‐question relevance: the study analyzed nursing‐home quality measures derived primarily from Minimum Data Set resident‐assessment data, and the causal question concerned nursing‐home care quality.

*Note:* Companion reports are combined. Outcomes, time horizons, causal targets, identification strategies, ML roles, and findings are reported in Table 3.

Abbreviations: AI, artificial intelligence; AMPATH, Academic Model Providing Access to Healthcare; BERT, Bidirectional Encoder Representations from Transformers; COMPOSER, COnformal Multidimensional Prediction Of SEpsis Risk; EHR, electronic health record; HIV, human immunodeficiency virus; HI‐FIVES, Helping Invested Family Members Improve Veteran's Experiences Study; ML, machine learning; STRATIFY, St. Thomas's Risk Assessment Tool in Falling Elderly Inpatients.

All five Category 1 studies met intervention‐role relevance. Among the ten Category 2 studies, eight met end‐user relevance, Liu et al. (2026) met educational relevance, and Barrett et al. (2025) met data‐and‐question relevance. Cho et al. (2021) also met intervention‐role relevance. Barrett et al. (2025) was the only study to qualify solely through data‐and‐question relevance. Study‐level causal targets, identification strategies, estimators, ML roles, and findings are summarized in Table 3. Detailed report‐level information is provided in Table S2 for Category 1 and Table S3 for Category 2.

**TABLE 3:** Causal and machine‐learning characteristics of the included studies.

Study (reports)	Causal target	Identification strategy	Estimator and substantive ML role	Main finding(s)
Category 1. ML within causal effect estimation				
Tran et al. (2016), Miles et al. (2019)	Tran: population mean risk of death or loss to follow‐up under three longitudinal LREC strategies and a controlled direct effect, through 450 days. Miles: direct and overall effects under exposure‐reallocation schemes with fixed clinic coverage, through 270 days.	Consistency, sequential exchangeability, and positivity. Tran assumed no interference; Miles used a stratified‐interference framework.	Longitudinal TMLE with Super Learner nuisance estimation; Miles extended TMLE to interference. ML was used within the primary causal estimator.	Tran estimated in‐care survival of 0.93 with immediate availability and enrolment, 0.87 with availability without enrolment, and 0.91 without availability at 450 days. Miles found direct benefit in all but one clinic and limited benefit from random reallocation, with some improvement under CD4‐based allocation.
Marafino et al. (2020)	Conditional and individualized effects of the Transitions Program on 30‐day nonelective readmission.	Conditional exchangeability given measured covariates, consistency, and overlap during phased implementation; inference accounted for hospital clustering.	Causal forest with propensity and outcome nuisance models and out‐of‐bag prediction. ML directly estimated patient‐level treatment effects and alternative targeting policies.	Estimated benefit was heterogeneous and did not align with predicted readmission risk. Effect‐based targeting was projected to prevent up to 4458 readmissions annually, compared with approximately 1246 under actual program targeting.
Langenberger et al. (2023)	Average and conditional effects of the electronic patient‐reported outcome alert intervention on 12‐month patient‐reported outcomes.	Random treatment assignment identified the intervention contrast; overlap and honest out‐of‐bag estimation supported the heterogeneous‐effect analysis.	Augmented inverse‐propensity‐weighted average effects and honest causal forests for conditional effects. ML was used in the primary effect‐estimation procedure.	Average effects were small. Prespecified subgroup contrasts suggested heterogeneity, although omnibus tests provided limited evidence of global treatment‐effect heterogeneity.
Moges et al. (2025a, 2025b)	Average and individualized effects of midwife‐led continuity care on low birth weight at birth.	Quasi‐experimental site comparison with covariate adjustment, propensity modeling, and representation balancing; formal identifying assumptions were only partly articulated.	Moges et al. (2025a) used causal classifiers, gradient boosting, and S‐, T‐, X‐, and R‐learners. Moges et al. (2025b) used counterfactual representation learning with Wasserstein‐ and maximum‐mean‐discrepancy balancing. ML was used in primary effect estimation.	Reported average‐effect estimates varied across methods, from 0.237 to 0.45, and individualized effects were also presented. The effect scale and its interpretation were not fully consistent across the companion reports.
Shepherd‐Banigan et al. (2020)	Heterogeneous effects of HI‐FIVES on cumulative days not at home and caregiver depressive symptoms at 12 months.	Randomization of the parent trial identified the treatment contrast; cross‐fold and alternative‐tree analyses were used to assess stability.	Model‐based recursive partitioning, matched causal‐tree sensitivity analysis, and random‐forest interaction analysis. ML was used to identify data‐adaptive treatment‐effect subgroups.	Lower caregiver burden identified a subgroup with a more favorable effect on days at home; lower baseline depressive symptoms identified a subgroup with slightly lower depressive‐symptom scores at follow‐up.
Category 2. Causal evaluation of an ML‐enabled intervention, tool, workflow, or targeting strategy				
Shimabukuro et al. (2017)	Effect of assignment to ML sepsis monitoring on hospital length of stay and in‐hospital mortality.	Computer‐generated patient‐level randomization with intention‐to‐treat group comparison.	Conventional randomized‐group comparisons; the ML classifier was a functional component of the monitored alert intervention rather than the causal estimator.	Mean hospital stay was shorter in the intervention group than in the control group (10.3 vs. 13.0 days), and in‐hospital mortality was 12.4 percentage points lower.
Liu et al. (2026)	Effect of access to an LLM pedagogical agent on nursing simulation‐design ratings, self‐efficacy, usability, and satisfaction after 2 months.	Random‐number‐table allocation; no allocation concealment or blinding. Selected age‐adjusted sensitivity analyses were reported.	Between‐group tests and selected covariance analyses; the knowledge‐grounded LLM generated scenarios, formative guidance, and on‐demand learning support.	The LLM group had higher simulation‐design ratings and higher scores in selected self‐efficacy domains. Usability did not differ, and satisfaction showed a ceiling effect.
Barwise et al. (2026)	Intention‐to‐treat effect of AI‐guided outreach on receipt of an in‐person interpreter and time to first interpreter during admission.	Randomized stepped‐wedge cluster crossover with adjustment for time period and nursing‐unit clustering.	Mixed‐effects modeling and a Cox model; an ML‐derived complexity ranking targeted secure‐chat outreach to bedside nurses.	Interpreter use was 44% versus 41% (adjusted OR 1.40, 95% CI 0.98–2.00). Time to first interpreter did not differ (HR 1.02, 95% CI 0.81–1.29).
Scott et al. (2025)	Intention‐to‐treat effect of visible septic‐shock decision support on early sepsis treatment, antibiotic timing, and shock within 24 h.	Randomized stepped‐wedge activation order with site and period adjustment.	Generalized linear and proportional‐hazards models; two ML prediction models generated the visible clinical decision‐support alert.	The primary treatment outcome was 39.0% versus 38.9% (adjusted OR 1.07). No material effect was found on shock or time to antibiotic.
Gallo et al. (2024)	Local effect of activating the deterioration alert and huddle on escalation in care among patients near the EDI threshold.	Sharp regression discontinuity, assuming continuity of potential outcomes and no threshold manipulation; balance, density, placebo, and bandwidth checks were reported.	Local linear regression with triangular kernels; the EDI prediction score triggered a nurse‐physician huddle.	At the threshold, the intervention was associated with a 10.4‐percentage‐point absolute reduction in the escalation outcome.
Cho et al. (2021)	Immediate level and post‐implementation trend changes in falls, fall injuries, and nursing prevention processes.	Controlled interrupted time series using matched intervention and control nursing units, with adjustment for patient and process characteristics.	Segmented time‐series models; a Bayesian‐network fall‐risk tool generated daily alerts and risk‐linked nursing care recommendations.	The controlled ITS reported a −0.10 coefficient for the post‐ versus pre‐intervention change in fall rates (SE 0.04, *p* = 0.014). There was no sustained trend effect or reduction in fall injuries, while targeted nursing interventions increased.
Boussina et al. (2024)	Causal impact of COMPOSER deployment on mortality, sepsis‐bundle compliance, and the 72‐h change in SOFA score.	Bayesian structural time‐series counterfactual model trained on a 705‐day pre‐period, with seasonal and confounder adjustment and posterior counterfactual intervals.	Causal Impact analysis; the COMPOSER deep‐learning model generated a nurse‐facing advisory with explanatory features.	Deployment was associated with a 1.9‐percentage‐point reduction in mortality, a 5.0‐percentage‐point increase in sepsis bundle compliance, and a 4% reduction in the average 72‐h change in SOFA score following sepsis onset.
Yang et al. (2024)	Differential change in median time to first nurse read for high‐scoring versus low‐scoring patient messages after implementation.	Difference‐in‐differences using low‐scoring messages as the comparison trend; pre‐period parallel trends were assessed visually.	Quantile‐regression difference‐in‐differences; a fine‐tuned BERT model produced a high‐acuity flag used to reorder the nurse message queue.	The estimated intervention‐attributable median reduction was 9 min for messages sent during business hours and 21 min for messages sent outside business hours.
Verma et al. (2024)	Effect of CHARTwatch implementation on non‐palliative in‐hospital death and care‐process outcomes.	Propensity‐score overlap weighting for pre‐post balance, supplemented by a contemporaneous difference‐in‐differences comparison with medical subspecialty units.	Weighted regression and difference‐in‐differences models; a time‐aware MARS deterioration model generated alerts and activated a high‐risk pathway.	The adjusted pre‐post risk ratio for non‐palliative death was 0.74. The controlled difference‐in‐differences risk ratio was 0.79 (95% CI 0.50–1.24), which included the null.
Barrett et al. (2025)	Difference‐in‐differences effects of adding the AI component to an EHR on 18 nursing‐home quality measures across 11 quarters.	Facility‐level difference‐in‐differences with mixed‐effects models; parallel trends and baseline comparability were evaluated for each outcome.	Outcome‐specific linear mixed‐effects models; hybrid learned algorithms generated resident alerts and facility‐level clinical metrics.	Sixteen of 18 measures showed statistically greater improvement in EHR‐plus‐AI facilities, and 11 of these also satisfied the reported parallel‐trends criterion. Among the latter, major falls decreased by 9% (95% CI −17, −1) and residents needing help with daily activities by 22% (95% CI −29, −15).

*Note:* Companion reports are combined. Causal‐target terminology follows the study authors when explicit; otherwise, reviewer‐charted intervention contrasts are reported.

Abbreviations: AI, artificial intelligence; BERT, Bidirectional Encoder Representations from Transformers; CD4, cluster of differentiation 4; CI, confidence interval; COMPOSER, COnformal Multidimensional Prediction Of SEpsis Risk; EDI, Epic Deterioration Index; EHR, electronic health record; HR, hazard ratio; HI‐FIVES, Helping Invested Family Members Improve Veteran's Experiences Study; ITS, interrupted time series; LLM, large language model; LREC, Low Risk Express Care; MARS, multivariate adaptive regression splines; ML, machine learning; OR, odds ratio; SE, standard error; SOFA, Sequential Organ Failure Assessment; TMLE, targeted maximum likelihood estimation.

### 3.3 Category 1: ML Within Causal Effect Estimation

Category 1 studies addressed longitudinal and spillover effects, treatment‐effect heterogeneity, and effect‐based targeting. Tran et al. (2016) and Miles et al. (2019) used targeted maximum likelihood estimation with Super Learner to evaluate alternative Low Risk Express Care strategies. Tran et al. estimated the highest survival under program availability with enrolment, while program availability without enrolment produced a small adverse controlled direct effect. Miles et al. found beneficial direct effects in all but one clinic and limited benefit from random reallocation, with some improvement under CD4‐based allocation.

The remaining studies focused on treatment‐effect heterogeneity. Marafino et al. (2020) found that the estimated benefit from a post‐discharge program did not align with the predicted readmission risk, whereas Langenberger et al. (2023) found little evidence of systematic heterogeneity. The Moges companion (Moges et al. 2025a, 2025b) reports the estimated average and individual effects of midwife‐led continuity care using distinct causal ML approaches, although the effect scale and interpretation were not reported consistently. Shepherd‐Banigan et al. (2020) identified exploratory subgroups defined by baseline caregiver characteristics.

### 3.4 Category 2: Causal Evaluation of an ML‐Enabled Intervention, Tool, Workflow, or Targeting Strategy

Four studies used individual‐level allocation or stepped‐wedge cluster randomization, with mixed findings. Shimabukuro et al. (2017) reported shorter hospital stay and lower mortality with ML sepsis monitoring. Liu et al. (2026) reported higher simulation‐design ratings and selected self‐efficacy scores, while Barwise et al. (2026) produced an imprecise estimate for interpreter use and Scott et al. (2025) found no material effect on the evaluated sepsis outcomes.

Among studies using threshold or time‐series designs, Gallo et al. (2024) estimated lower escalation in care near the deterioration‐alert threshold. Cho et al. (2021) found a greater immediate fall‐rate reduction and increased risk‐targeted nursing interventions, but no sustained trend effect or reduction in fall‐related injuries. Boussina et al. (2024) reported lower mortality together with improvements in sepsis bundle compliance and Sequential Organ Failure Assessment change.

In the remaining controlled pre‐post or difference‐in‐differences evaluations, Yang et al. (2024) reported shorter nurse message‐review times. Verma et al. (2024) found a favorable pre‐post mortality estimate, but the controlled difference‐in‐differences estimate was imprecise and included no effect. Barrett et al. (2025) reported favorable estimates for most nursing‐home quality measures, although only 11 outcomes met or conditionally met the reported parallel‐trends criterion.

### 3.5 Cross‐Study Patterns in Causal Specification, ML Role, and Estimated Effects

Causal specification differed systematically between the categories. Category 1 studies generally named formal causal estimands, including average, subgroup, individual‐level, direct, overall, and alternative‐allocation effects. Category 2 studies more often defined practical targets linked to the design. Examples included the effect of assignment, an effect near an alert threshold, a change after implementation, a comparison with predicted outcomes without deployment, or a change relative to a comparison group. Formal estimand terminology was not always used, but the intervention, comparator, outcome, target population, and time horizon were explicitly specified. Identification likewise differed by design. Category 1 trial‐based analyses relied on randomization, whereas observational analyses depended on exchangeability, positivity, and, where relevant, assumptions about interference. Category 2 studies relied on randomization or design‐specific assumptions, including local comparability and absence of manipulation for threshold designs and credible counterfactual trends for time‐based designs. Reporting of identification assumptions was uneven, particularly across the Moges companion reports.

The substantive ML role also differed consistently. In Category 1, ML directly estimated treatment effects or modeled the outcome and treatment assignment needed to calculate them. In Category 2, the trained ML model generated a prediction, ranking, alert, risk category, or educational response that guided prioritization, monitoring, escalation, communication, education, or care planning. The evaluated intervention therefore included the model and its surrounding human and organizational workflow rather than the algorithm in isolation.

Category 1 findings focused on heterogeneity in benefit, targeting decisions, and alternative allocation strategies rather than on a common intervention effect. Among the four randomized or allocated Category 2 evaluations, two reported favorable clinical or selected educational outcomes, one produced an imprecise estimate compatible with no effect, and one found no material effect. Among the six non‐randomized evaluations, five reported at least one favorable controlled estimate with an interval or test excluding no effect. The controlled CHARTwatch estimate also favored the intervention, but its 95% confidence interval included no effect. Accordingly, the findings were not treated as estimates of a common effect because interventions, outcomes, populations, and causal targets differed.

## 4 Discussion

### 4.1 Principal Findings

This review identified 15 studies represented by 17 reports: five used ML within causal effect estimation and ten evaluated ML‐enabled interventions, tools, workflows, or targeting strategies. Collectively, these studies formed a small, heterogeneous, and geographically concentrated evidence base, with mixed findings across distinct causal questions. These findings apply only to studies meeting the review's explicit causal threshold and should not be extrapolated to the wider nursing ML literature.

### 4.2 Two Distinct Roles of ML

In Category 1, ML supported causal effect estimation by modeling potential outcomes, treatment‐effect functions, or nuisance functions after the causal estimand and identification strategy had been specified. Flexible ML may strengthen estimation, but it does not itself establish identification (Dang et al. 2023; Feuerriegel et al. 2024). Conversely, in Category 2, ML was a functional component of the intervention or workflow, generating outputs that guided subsequent action. The causal target therefore concerned the implemented model‐plus‐workflow package rather than algorithm performance in isolation. The two categories address different research questions, not different levels of methodological quality, and Category 2 effects may reflect the model, implementation strategy, professional response, organizational capacity, or their interaction (Magrabi et al. 2019).

Heterogeneous‐effect studies further showed that predicted risk need not align with estimated benefit. Marafino et al. (2020) illustrated this directly, whereas Langenberger et al. (2023) and Shepherd‐Banigan et al. (2020) provided less definitive subgroup evidence. Data‐adaptive subgroup findings therefore require adequate sample support, clear distinction between prespecified and exploratory analyses, and independent confirmation before clinical use (Kent et al. 2020).

### 4.3 Causal Specification and Identification

Across both categories, causal interpretation required separation of the causal target, identification strategy, and estimator. Category 1 reports more often named formal average, heterogeneous, direct, or allocation‐related effects, whereas Category 2 reports more often specified design‐linked targets such as assignment, threshold, or change‐in‐change effects. Formal use of the term estimand was not required, but the intervention or exposure, comparator, outcome, target population, time horizon, effect measure, and causal contrast had to be clear; naming a causal method alone was insufficient (Dahabreh and Bibbins‐Domingo 2024; Dang et al. 2023).

Identification assumptions were design‐specific. Randomized studies relied on the allocation process, observational effect‐estimation studies on exchangeability and related assumptions, regression discontinuity on continuity and absence of manipulation, difference‐in‐differences on credible pre‐intervention trends, and time‐series approaches on a defensible counterfactual trajectory. Reporting remained uneven, particularly across the Moges companion reports, whereas Miles et al. explicitly incorporated interference and alternative allocation strategies. This variation supports the review's requirement for both a defined causal target and a corresponding identification strategy.

### 4.4 ML‐Enabled Systems and Nursing or Midwifery Practice

The Category 2 findings reinforce established AI evaluation guidance. SPIRIT‐AI, CONSORT‐AI, and DECIDE‐AI emphasize intended use, users, outputs, human–AI interaction, workflow integration, and implementation context (Cruz Rivera et al. 2020; Liu et al. 2020; Vasey et al. 2022). This is directly relevant to nursing and midwifery because the effects of an AI‐enabled system depend partly on how it is integrated into care delivery and how users respond to it (Magrabi et al. 2019).

Importantly, the findings also support distinguishing nurse‐facing, nurse‐implemented, and nurse‐led roles. In 8 of the 10 Category 2 studies, nursing professionals occupied the action point between model output and downstream action. Evaluation should therefore include nursing‐process outcomes alongside patient outcomes (Mebrahtu et al. 2021). Depending on the workflow, relevant balancing outcomes may include response timeliness, workload, alert burden, overrides, safety, and equity. Barrett et al. (2025) was the only study to qualify through data‐and‐question relevance, indicating that explicitly causal analyses using nursing data as the primary analytic source remain rare.

### 4.5 Relation to Previous Reviews

Previous nursing‐focused syntheses mapped ML applications and model evaluation (Choi et al. 2025), methodological and reporting characteristics and clinical implementation (Kim and Kim 2025), prediction modeling using nursing data in intensive care (Kim et al. 2025), and implementation of deterioration‐prediction systems (Peelen et al. 2021). In essence, the present review differed by requiring an explicit causal estimand, corresponding identification strategy, substantive ML role, and operational nursing or midwifery relevance, while distinguishing ML used within effect estimation from ML embedded in the intervention being evaluated. This narrower lens produced a smaller but more causally specific evidence map.

### 4.6 Methodological and Reporting Implications

Accordingly, future studies should begin with a clearly stated causal question and define the intervention or exposure, comparator, outcome, target population, time horizon, and effect measure before specifying the identification strategy, its assumptions, the estimator, and the ML method. This sequence follows the causal roadmap described by Dang et al. (2023). Design selection should reflect the assignment mechanism, available data, and causal target; no randomized or quasi‐experimental design label alone guarantees causal validity (Dang et al. 2023; Handley et al. 2018).

Longitudinal g‐methods may be required for time‐varying confounding and interference‐aware approaches when outcomes depend on other units' exposures (Hernán and Robins 2020; Miles et al. 2019). Category 1 reports should distinguish average from heterogeneous targets, explain the ML function, report relevant diagnostics, and quantify uncertainty (Kent et al. 2020; Wager and Athey 2018). Category 2 reports should describe the pathway from model input and output to professional response and downstream action, including adherence, overrides, implementation fidelity, error cases, and balancing outcomes (Liu et al. 2020; Magrabi et al. 2019; Vasey et al. 2022).

### 4.7 Strengths and Limitations

Strengths included expanded searches across eight electronic sources with backward citation searching, independent piloting, screening, and full‐text reassessment by two reviewers, study‐level linkage of companion reports, and explicit operational criteria for causal, ML, and nursing or midwifery relevance.

Nevertheless, limitations include the absence of prospective protocol registration, English‐language full‐text eligibility, and the possibility that relevant studies were missed because causal methods, ML systems, and nursing workflows are inconsistently described and indexed. Eligibility also depended on sufficiently explicit reporting of the causal estimand and identification strategy in at least one retrieved full report, and the two‐category framework and nursing or midwifery relevance pathways were author‐developed operational definitions.

No formal risk‐of‐bias or methodological quality appraisal was undertaken, so the validity or certainty of individual effect estimates was not assessed. No formal inter‐reviewer agreement statistic was calculated. The corpus was small and heterogeneous, with nine of 15 studies conducted in the United States, which precluded quantitative synthesis and limited transferability across populations, staffing models, workflows, and implementation contexts.

## 5 Conclusions

Taken together, the eligible evidence showed that ML either supported causal effect estimation or formed part of an ML‐enabled intervention or workflow whose causal effect was evaluated. Both roles require explicit estimands and credible identification, while implemented systems also require clear specification of the surrounding workflow. Consistent with established AI evaluation guidance, future studies should evaluate model‐plus‐workflow effects on patient outcomes and nursing processes, including workload, safety, equity, and unintended consequences.

## Patient Consent Statement

Patient consent was not required because this study did not involve human participants or identifiable patient data.

## Author Contributions

**Dimitrios Kosmidis:** conceptualization, investigation, writing – original draft, methodology, validation, visualization, writing – review and editing, data curation, supervision, project administration, formal analysis, resources, software. **Georgios Vlachopoulos:** writing – original draft, writing – review and editing, visualization, data curation. **Abdulqadir J. Nashwan:** visualization, writing – review and editing, writing – original draft, data curation, validation. **Rafael Lima Rodrigues de Carvalho:** visualization, writing – review and editing, data curation, validation, writing – original draft. **Breno Santos Araujo:** writing – review and editing, visualization, data curation, validation, writing – original draft. **Dimitra Armeni:** data curation, methodology, writing – review and editing, resources, investigation, formal analysis. **Eleni Sertaridou:** writing – review and editing, writing – original draft, visualization, data curation, resources, supervision. **Sotiria Koutsouki:** conceptualization, methodology, writing – original draft, writing – review and editing, investigation, data curation, project administration, supervision, validation, visualization, formal analysis.

## Funding

The authors have nothing to report.

## Ethics Statement

Ethics approval was not required because this scoping review used data from previously published studies and did not involve human participants, human tissue, or identifiable personal data.

## Conflicts of Interest

The authors declare no conflicts of interest.

## Supporting information


**Box S1:** Operational criteria for nursing and midwifery relevance.

**Data S1:** Supporting information.

**Table S1:** Complete database search strategies and records retrieved.

**Table S2:** Category 1 report‐level causal specification and estimation details.
**Table S3:** Category 2 report‐level ML workflow, assignment, and identification details.

## Data Availability

The data that supports the findings of this study are available in the supporting information of this article.
