# Supplementary material for: Machine Learning in Causal Effect Estimation and Causal Evaluation of ML‐Enabled Interventions in Nursing‐Related Research: A Scoping Review
Source: Nurs Health Sci. 2026 Oct 7;28(4):e70431. doi: 10.1111/nhs.70431 (PMC13645453; doi:10.1111/nhs.70431)
Supplement: Supplementary file 1 [file NHS-28-e70431-s001.docx]

| **Supplementary Table S2. Category 1 report-level causal specification and estimation details** | | | | |
| --- | --- | --- | --- | --- |
| **Study/report** | **Causal estimand** | **Identification strategy and assumptions/checks** | **Estimator and detailed ML function** | **Study-reported limitations** |
| Tran et al. (2016) Companion report to Miles et al. (2019) | Outcome(s): Death or loss to follow-up, expressed as in-care survival Target population and time horizon: Clinically stable patients eligible for task shifting. Up to 450 days after initial eligibility. Author-specified causal target or estimand: Population mean counterfactual outcome under three fixed interventions and the controlled direct effect of programme availability Review-standardised causal contrast: Joint effect of availability and enrolment, plus direct effect of availability while preventing enrolment | Identification strategy: Longitudinal g-formula identification under consistency, sequential exchangeability, positivity, and no interference The paper explicitly maps causal model, target parameters, identifiability, positivity, and the observed-data statistical estimand | Estimator or analysis: Longitudinal targeted maximum likelihood estimation ML method: Super Learner for longitudinal nuisance functions Counterfactual survival estimates and causal contrasts under fixed intervention strategies. Estimate the effect of programme availability and enrolment and demonstrate the targeted-learning roadmap | Observational sequential-exchangeability assumptions, possible positivity limitations, complex longitudinal exposure definitions, and no-interference assumption later revisited by Miles |
| Miles et al. (2019) Companion report to Tran et al. (2016) Clinic-specific analytic subsets of the Tran et al. cohort | Outcome(s): Death or loss to follow-up within 270 days Target population and time horizon: Clinically stable adults receiving HIV care and eligible for task shifting. 270 days from a clinic-specific baseline period Author-specified causal target or estimand: Direct effects and overall effects under specified exposure-reallocation schemes Review-standardised causal contrast: Effect of individual task-shifting enrolment and alternative allocation rules while holding the clinic's observed proportion enrolled fixed | Identification strategy: Nonparametric identification under stratified interference with conditional exchangeability and positivity Explicit structural model, stratified-interference assumption, exchangeability, positivity, consistency, and restriction to the observed exposure proportion | Estimator or analysis: Targeted maximum likelihood estimation with clinic-specific Super Learner nuisance models and marginal structural summaries ML method: Super Learner library including generalised linear models, least absolute shrinkage and selection operator regression, random forest, gradient boosting, and support-vector machines Detailed ML function: Direct and overall effect estimates under alternative exposure-allocation rules. Evaluate whether nurse task shifting was beneficial and whether alternative assignment rules could improve outcomes | Possible unmeasured confounding, dependence on the assumed interference structure, only one observed exposure proportion per clinic, and limited extrapolation to different programme coverage levels |
| Marafino et al. (2020) | Outcome(s): Non-elective readmission within 30 days Target population and time horizon: Adults discharged alive from participating hospitals. Thirty days after discharge Author-specified causal target or estimand: Conditional average treatment effect of the Transitions Program on readmission Review-standardised causal contrast: Difference in 30-day readmission risk under assignment to the Transitions Program versus no assignment, conditional on patient covariates | Identification strategy: Conditional exchangeability given measured covariates, consistency, and overlap during phased implementation The appendix states unconfoundedness and related assumptions. Propensity and outcome models supported orthogonalisation, and inference accounted for hospital clustering | Estimator or analysis: Causal forest with 8,000 trees, hospital clustering, out-of-bag prediction, and nuisance-model orthogonalisation ML method: Causal forest and auxiliary propensity and outcome models Patient-level predicted CATEs and alternative treatment-allocation strategies. Identify patients with the largest expected benefit rather than the highest outcome risk | Unverifiable no-unmeasured-confounding assumption, need for intervention-era data, some variables unavailable prospectively, and ethical risks of deprioritising high-risk patients |
| Langenberger et al. (2023) | Outcome(s): EQ-5D-5L, EQ-VAS, HOOS-PS or KOOS-PS, PROMIS depression, and PROMIS fatigue Target population and time horizon: Adults undergoing hip or knee arthroplasty. Twelve months after discharge Author-specified causal target or estimand: Average treatment effect and conditional average treatment effects Review-standardised causal contrast: Mean and subgroup-specific effect of assignment to the ePROM alert intervention versus control on 12-month PROM outcomes | Identification strategy: Patient randomisation identified the intervention contrast Random allocation, propensity-score overlap assessment, honest forest estimation, and out-of-bag prediction | Estimator or analysis: Augmented inverse-propensity-weighted ATE estimation and honest causal forests for CATEs ML method: Causal forest using the grf framework Patient-level and subgroup CATE estimates. Analytic identification of subgroups that may benefit more from the monitoring intervention | Post hoc secondary analysis, potential instability of subgroup findings, adherence and alert exposure varied, and some subgroup variables were post-randomisation |
| Moges et al. (2025a) Companion analytic report; study eligibility established by Moges et al. (2025b) | Outcome(s): Low birth weight below 2,500 g Target population and time horizon: Low-risk pregnant women attending selected hospitals. Birth outcome after enrolment during August 2019 to September 2020 Author-specified causal target or estimand: Average treatment effect and individual treatment effects of MLCC on low birth weight Review-standardised causal contrast: Effect of MLCC versus other professional care on the probability of low birth weight | Identification strategy: The same underlying quasi-experimental site comparison as Moges et al. (2025b), analysed using covariate-based causal-ML estimators. This companion report did not independently restate the full identification argument. Treated and control potential outcomes were defined, and covariate-based causal models were applied. Exchangeability and positivity were not explicitly articulated in this report. | Estimator or analysis: Causal KNN and causal random forest for modelling, gradient boosting for effect estimation, and S-, T-, X-, and R-learners ML method: Causal KNN, causal random forest, XGBoost, and meta-learners ATE and ITE predictions. Estimate overall and individualised benefit of MLCC and explore alternative targeting | Quasi-experimental allocation, potential residual confounding, missing maternal and fetal variables, incomplete real-world records, and unclear interpretation of some reported effect metrics. The effect scale and interpretation were not described consistently across the companion reports. Study eligibility was established through the fuller identification account in Moges et al. (2025b). |
| Moges et al. (2025b) Qualifying report for the underlying study; companion to Moges et al. (2025a) | Outcome(s): Low birth weight below 2,500 g Target population and time horizon: Low-risk pregnant women in the selected hospitals. Birth outcome after enrolment during August 2019 to September 2020 Author-specified causal target or estimand: Average treatment effect and heterogeneous or individual-level effects of MLCC on low birth weight Review-standardised causal contrast: Effect of MLCC versus other professional care on low-birth-weight risk | Identification strategy: Quasi-experimental site comparison with propensity modelling and representation balancing. This report provided the fuller account of the non-randomised identification basis for the underlying study. The approach sought balance between treated and control representations and addressed selection bias and limited overlap. Formal labels and a complete enumeration of the identifying assumptions were only partly articulated. | Estimator or analysis: Counterfactual regression with Wasserstein distance and maximum mean discrepancy, supported by Bayesian Ridge propensity modelling ML method: Counterfactual convolutional neural network, CFR-WASS, CFR-MMD, and Bayesian Ridge Counterfactual outcomes, ATE and heterogeneous-effect estimates, with PEHE reported as a model-performance metric. Estimate overall and individual-level MLCC effects | Quasi-experimental selection, incomplete covariate capture, limited causal-assumption reporting, and uncertain interpretation of PEHE and the reported effect scale in real data. The effect scale and interpretation were not described consistently across the companion reports |
| Shepherd-Banigan et al. (2020) | Outcome(s): Cumulative days not at home and caregiver depressive symptoms Target population and time horizon: Informal caregivers of community-dwelling older veterans with functional or cognitive impairment. Twelve months after randomisation Author-specified causal target or estimand: Heterogeneous treatment effects across caregiver and patient characteristics Review-standardised causal contrast: Subgroup-specific effect of HI-FIVES versus usual care on the two 12-month outcomes | Identification strategy: Randomisation of the parent trial identified the treatment contrast Computer-generated stratified randomisation. The secondary analysis used cross-fold checks and sensitivity methods to limit overfitting | Estimator or analysis: Model-based recursive partitioning, matched causal tree sensitivity analysis, and random-forest interaction analysis ML method: Model-based recursive partitioning and random forest Data-adaptive subgroups and subgroup treatment-effect estimates. Identify caregiver subgroups for whom the intervention may be more or less beneficial | Small sample for ML subgroup discovery, post hoc analysis, unstable single-tree partitions, limited external validation, and outcome-distribution constraints |
| ***Note.*** Companion reports are shown separately when they contributed distinct causal estimands, assumptions, or ML estimators. Eligibility of the Moges study was established by Moges et al. (2025b); Moges et al. (2025a) was retained as a companion analytic report. Full references for the included reports are provided in the reference list of the main manuscript. **Abbreviations:** ATE, average treatment effect; CATE, conditional average treatment effect; CFR-MMD, counterfactual regression with maximum mean discrepancy; CFR-WASS, counterfactual regression with Wasserstein distance; HI-FIVES, Helping Invested Family Members Improve Veteran’s Experiences Study; HIV, human immunodeficiency virus; ITE, individual treatment effect; KNN, k-nearest neighbours; ML, machine learning; MLCC, midwife-led continuity care; PEHE, precision in estimation of heterogeneous effects; PROM, patient-reported outcome measure; TMLE, targeted maximum likelihood estimation. | | | | |

| **Supplementary Table S3. Category 2 report-level ML workflow, assignment, and identification details** | | | | | |
| --- | --- | --- | --- | --- | --- |
| **Study/report** | **Unit of assignment or exposure and unit of analysis** | **ML output and triggered or guided action** | **Intended users, workflow integration, and relevance pathway** | **Identification strategy and assumptions/checks** | **Implementation and interpretive cautions** |
| Shimabukuro et al. (2017) | Unit of assignment or exposure: Patient randomisation. Unit of analysis: Patients. | ML output: Hourly sepsis risk score and telephone alert at threshold 80 Triggered or guided action: The charge nurse evaluated the patient and followed the standard severe-sepsis assessment and escalation pathway | Intended users: Charge nurses and the clinical care team Workflow integration: The model monitored patients continuously. Alerts were communicated by telephone to the charge nurse without treatment recommendations Relevance pathway: End-user relevance | Identification strategy: Computer-generated patient-level randomisation Concealed backend allocation before alerting. Assignment became apparent when an alert was generated. Intention-to-treat group comparison | Small sample, single centre, short trial duration, incomplete blinding after alerts, and limited generalisability |
| Liu et al. (2026) | Unit of assignment or exposure: Nurse-participant allocation. Unit of analysis: Nurse participants. | ML output: Generated scenarios, formative guidance, question answering, and on-demand learning support Triggered or guided action: Nurses used the agent to design and implement ICU simulation teaching activities. | Intended users: ICU nurse learners and simulation instructors Workflow integration: Human-machine collaborative learning combined instructor guidance, agent support, and learner activity. Relevance pathway: Educational | Identification strategy: Random-number-table assignment provided the main identification strategy, although allocation concealment and blinding were not used External generation of the allocation sequence. No concealment or blinding. Age-adjusted sensitivity analysis was reported for selected outcomes. | Small single-centre sample, subjective outcomes, no long-term or objective performance outcomes, no blinding, possible contamination, and unequal learning exposure |
| Barwise et al. (2026) | Unit of assignment or exposure: Inpatient-unit randomisation. Unit of analysis: Hospital admissions. | ML output: Daily ranked list of eligible complex patients Triggered or guided action: Language services used the ranking to target secure-chat prompts to bedside nurses | Intended users: Language-services personnel and bedside nurses. Workflow integration: The ML output initiated a language-services-to-nurse communication pathway. The nurse then contacted language services to arrange the interpreter Relevance pathway: End-user relevance | Identification strategy: Randomised stepped-wedge crossover with adjustment for time period and clustering Random sequence of unit crossover, period adjustment, and nursing-unit random effects | Single centre, limited interpreter availability, occasional technical failures, variable unit workflows, and no patient-centred clinical outcomes |
| Scott et al. (2025) | Unit of assignment or exposure: Emergency-department site randomisation. Unit of analysis: Emergency-department encounters. | ML output: High-risk septic-shock best-practice alert Triggered or guided action: The alert prompted provider reassessment and consideration of sepsis evaluation and treatment | Intended users: Physicians, nurse practitioners, and physician assistants Workflow integration: The visible alert appeared in the EHR. Advanced-practice nurses were among intended recipients. Bedside nurses could initiate sepsis evaluation but did not receive the study alert directly Relevance pathway: End-user relevance | Identification strategy: Randomised stepped-wedge activation order with period and site adjustment Cluster randomisation, fixed effects for the stepped-wedge structure, and adjusted analyses for observed arm imbalance | Four sites in one network, infrequent alerts, limited power, strong pre-existing sepsis care, cluster imbalance, and direct alerts not sent to bedside nurses |
| Gallo et al. (2024) | Unit of assignment or exposure: Exposure at the Epic Deterioration Index threshold. Unit of analysis: Hospital admissions. | ML output: EDI risk score and threshold alert Triggered or guided action: Nurse and physician conducted a structured huddle, reviewed causes, and considered preventive action | Intended users: Bedside nurses and physicians Workflow integration: The alert activated a collaborative nurse-physician assessment workflow Relevance pathway: End-user relevance | Identification strategy: Regression discontinuity using the EDI threshold as a pseudorandom assignment mechanism Continuity of potential outcomes, no manipulation of the running variable, covariate balance, McCrary test, placebo analysis, and bandwidth sensitivity analyses | Local estimate may not generalise away from the threshold, possible post-treatment influence on maximum EDI score, silent test alerts and comfort-care misclassification, and single-centre setting |
| Cho et al. (2021) | Unit of assignment or exposure: Matched nursing-unit allocation. Unit of analysis: Nursing-unit time periods and admissions. | ML output: Daily dichotomised fall-risk status and risk-factor-linked care recommendations Triggered or guided action: Nurses reviewed the risk output, could override it, and selected targeted fall-prevention interventions | Intended users: Bedside nurses and nursing-unit leadership Workflow integration: Predictions were integrated into the EHR and nursing care-planning screen with education and unit champions Relevance pathway: End-user relevance and intervention-role relevance | Identification strategy: Controlled interrupted time series with matched units and segmented analysis Concurrent control units, baseline matching, adjustment for patient characteristics and process measures, and explicit assessment of immediate and ongoing effects | Non-randomised unit allocation, single hospital, concurrent staffing-policy changes, rare outcomes, and possible residual unit-level confounding |
| Boussina et al. (2024) | Unit of assignment or exposure: Pre-deployment versus post-deployment time period. Unit of analysis: Patients and monthly outcome series. | ML output: Risk alert with the model's principal reasons Triggered or guided action: The nurse reviewed the alert and could notify the physician immediately or use secure chat | Intended users: Emergency-department nurses and physicians Workflow integration: The best-practice advisory appeared when nurses opened the chart. Nursing staff participated in iterative implementation and alert refinement Relevance pathway: End-user relevance | Identification strategy: Bayesian structural time-series model trained on a 705-day pre-period with confounder and seasonal adjustment Time-invariant regression effects, independence of the control-series response from the intervention, residual quantile-quantile and autocorrelation checks, and posterior counterfactual intervals | Non-randomised pre-post design, short post period, possible concurrent changes, single health system, abrupt implementation assumptions, and uncertain longer-term sustainability |
| Yang et al. (2024) | Unit of assignment or exposure: Implementation period and message-score group. Unit of analysis: Patient messages. | ML output: Binary High-acuity flag, later supplemented by a 0-100 score Triggered or guided action: Nurses sorted the queue by the AI output, reviewed high-priority messages first, and escalated urgent cases | Intended users: Registered-nurse care coordinators Workflow integration: The model output was integrated as an EHR display column within the existing two-stage triage and action workflow Relevance pathway: End-user relevance | Identification strategy: Difference-in-differences with low-scoring messages as the comparison trend Visual assessment of pre-period parallel trends and sensitivity to business-hours and off-hours message timing | Concurrent workflow initiatives, rapid growth in message volume and staffing, non-randomised design, incomplete quantitative measurement of individual nurse adoption, and single-system evaluation |
| Verma et al. (2024) | Unit of assignment or exposure: Time period and hospital unit. Unit of analysis: Hospital admissions. | ML output: High, medium, or low risk categories with alerts Triggered or guided action: High-risk alerts triggered physician reassessment within one hour, increased nurse vital-sign monitoring, and possible palliative-care consultation | Intended users: Physicians, charge nurses, bedside nurses, and palliative-care teams Workflow integration: Predictions were communicated by paging and email. Charge nurses used twice-daily risk information to support nurse-patient assignment and monitoring Relevance pathway: End-user relevance | Identification strategy: Propensity-score overlap weighting for pre-post comparability plus a contemporaneous difference-in-differences comparison Measured baseline balance after weighting, contemporaneous control units, exclusion of COVID-19 and influenza, and sensitivity analyses. Pandemic-related residual confounding remained possible | No randomised control, pandemic timing, possible residual confounding, limited statistical power, unplanned addition of subspecialty comparators, and single-centre implementation |
| Barrett et al. (2025) | Unit of assignment or exposure: Organisational adoption of Clinical Advanced Insights, self-selected at the nursing-home level. Unit of analysis: Facility-quarters. | ML output: Resident-level alerts and facility-level clinical metrics Triggered or guided action: Clinical staff used the output for proactive monitoring, medication review, and multidisciplinary care modification | Intended users: Nursing-home clinical staff and multidisciplinary teams Workflow integration: Outputs were incorporated into daily monitoring, medication review, and multidisciplinary care modification Relevance pathway: Data-and-question relevance | Identification strategy: Difference-in-differences with linear mixed-effects models and baseline covariate adjustment Parallel trends assessed for each outcome, facility comparability assessed, and covariates selected using standardised mean differences and multicollinearity checks | Organisational self-selection, possible unmeasured confounding, unverifiable counterfactual trends, limited turnover data, facility heterogeneity, and restricted generalisability |
| ***Note.*** ML was a functional component of the intervention, tool, workflow, or targeting strategy rather than the causal estimator. Full references for the included reports are provided in the reference list of the main manuscript. **Abbreviations:** AI, artificial intelligence; COVID-19, coronavirus disease 2019; EDI, Epic Deterioration Index; EHR, electronic health record; ICU, intensive care unit; ML, machine learning. | | | | | |
