# Supplementary material for: Machine Learning in Causal Effect Estimation and Causal Evaluation of ML‐Enabled Interventions in Nursing‐Related Research: A Scoping Review
Source: Nurs Health Sci. 2026 Oct 7;28(4):e70431. doi: 10.1111/nhs.70431 (PMC13645453; doi:10.1111/nhs.70431)
Supplement: Supplementary file 2 [file NHS-28-e70431-s002.docx]

**Supplementary Table S1. Complete database search strategies and records retrieved**

| **Database** | **Limits, filters, and fields** | **Records retrieved** |
| --- | --- | --- |
| **PubMed** | Search date: 11 June 2026 Date range: 1 January 2015-11 June 2026 Fields: title/abstract plus nursing-, midwifery-, and health-personnel MeSH terms Language filter: none | **848** |
| **Complete search strategy**  (  "causal inference"[tiab] OR  "causal effect*"[tiab] OR  "causal discovery"[tiab] OR  "causal machine learning"[tiab] OR  "causal artificial intelligence"[tiab] OR  counterfactual*[tiab] OR  "potential outcome*"[tiab] OR  "structural causal model*"[tiab] OR  "causal graph*"[tiab] OR  "directed acyclic graph*"[tiab] OR  DAG[tiab] OR  "PC algorithm*"[tiab] OR  "constraint-based causal discovery"[tiab] OR  "target trial emulation"[tiab] OR  "target trial"[tiab] OR  "emulated trial"[tiab] OR  "interrupted time series"[tiab] OR  "regression discontinuity"[tiab] OR  "regression discontinuity design*"[tiab] OR  "instrumental variable*"[tiab] OR  "instrumental variable analysis"[tiab] OR  "synthetic control*"[tiab] OR  "synthetic control method*"[tiab] OR  "g-computation"[tiab] OR  "g computation"[tiab] OR  "g formula"[tiab] OR  "g-formula"[tiab] OR  "parametric g-formula"[tiab] OR  "targeted maximum likelihood"[tiab] OR  "targeted maximum likelihood estimation"[tiab] OR  "targeted minimum loss"[tiab] OR  "targeted learning"[tiab] OR  TMLE[tiab] OR  "doubly robust"[tiab] OR  "doubly robust estimation"[tiab] OR  "doubly robust estimator*"[tiab] OR  "augmented inverse probability"[tiab] OR  AIPW[tiab] OR  "propensity score*"[tiab] OR  "inverse probability weight*"[tiab] OR  "inverse probability weighting"[tiab] OR  "marginal structural model*"[tiab] OR  "difference-in-differences"[tiab] OR  "difference in differences"[tiab] OR  "difference-in-difference"[tiab] OR  "difference in difference"[tiab] OR  "Bayesian structural time series"[tiab] OR  CausalImpact[tiab] OR  "causal impact"[tiab] OR  "stepped wedge"[tiab] OR  "quasi-experiment*"[tiab] OR  "quasi experiment*"[tiab] OR  "natural experiment*"[tiab] OR  "treatment effect*"[tiab] OR  "intervention effect*"[tiab] OR  "average treatment effect*"[tiab] OR  "conditional average treatment effect*"[tiab] OR  "heterogeneous treatment effect*"[tiab] OR  "individual treatment effect*"[tiab] OR  "individualized treatment effect*"[tiab] OR  ATE[tiab] OR  CATE[tiab] OR  "causal forest*"[tiab] OR  "causal tree*"[tiab] OR  "generalized random forest*"[tiab] OR  "meta-learner*"[tiab] OR  metalearner*[tiab] OR  "S-learner*"[tiab] OR  "T-learner*"[tiab] OR  "X-learner*"[tiab] OR  "R-learner*"[tiab] OR  "S learner*"[tiab] OR  "T learner*"[tiab] OR  "X learner*"[tiab] OR  "R learner*"[tiab]  )  **AND**  (  "machine learning"[tiab] OR  "machine learning model*"[tiab] OR  "artificial intelligence"[tiab] OR  "artificial intelligence model*"[tiab] OR  "deep learning"[tiab] OR  "neural network*"[tiab] OR  "artificial neural network*"[tiab] OR  "Bayesian network*"[tiab] OR  "random forest*"[tiab] OR  "gradient boosting"[tiab] OR  XGBoost[tiab] OR  transformer*[tiab] OR  BERT[tiab] OR  "large language model*"[tiab] OR  LLM[tiab] OR  LLMs[tiab] OR  "generative artificial intelligence"[tiab] OR  "generative AI"[tiab] OR  ChatGPT[tiab] OR  "natural language processing"[tiab] OR  NLP[tiab] OR  "predictive model*"[tiab] OR  "prediction model*"[tiab] OR  "clinical prediction model*"[tiab] OR  "risk prediction"[tiab] OR  "risk stratification"[tiab] OR  "clinical decision support"[tiab] OR  "clinical decision support system*"[tiab] OR  "computerized clinical decision support"[tiab] OR  "computerised clinical decision support"[tiab] OR  "decision support"[tiab] OR  CDSS[tiab] OR  alert*[tiab] OR  "early warning"[tiab] OR  "early warning score*"[tiab] OR  "clinical deterioration"[tiab] OR  "deterioration index"[tiab] OR  "electronic health record algorithm*"[tiab] OR  "EHR algorithm*"[tiab]  )  **AND**  (  nurs*[tiab] OR  "Nursing"[Mesh] OR  "Nurses"[Mesh] OR  "Nursing Care"[Mesh] OR  "Nursing Staff"[Mesh] OR  "Nurse Practitioners"[Mesh] OR  "Nursing Informatics"[Mesh] OR  midwif*[tiab] OR  "Midwifery"[Mesh] OR  "nursing care"[tiab] OR  "nursing workflow"[tiab] OR  "nursing documentation"[tiab] OR  "nursing intervention*"[tiab] OR  "nurse-led"[tiab] OR  "nurse led"[tiab] OR  "nurse-facing"[tiab] OR  "nurse facing"[tiab] OR  "nurse-implemented"[tiab] OR  "nurse implemented"[tiab] OR  "nurse alert*"[tiab] OR  "nursing alert*"[tiab] OR  "care delivery"[tiab] OR  "care workflow"[tiab] OR  "clinical workflow"[tiab] OR  "patient monitoring"[tiab] OR  triage[tiab] OR  "escalation of care"[tiab] OR  bedside[tiab] OR  ward[tiab] OR  wards[tiab] OR  inpatient*[tiab] OR  "Health Personnel"[Mesh] OR  "Allied Health Personnel"[Mesh]  )  **AND**  (  "2015/01/01"[pdat] : "2026/06/11"[pdat]  ) | | |
| **Scopus** | Search date: 11 June 2026 Publication years: 2015-2026 Fields: title, abstract, and keywords (TITLE-ABS-KEY) Language filter: none | **1,385** |
| **Complete search strategy**  (  TITLE-ABS-KEY(  "causal inference" OR  "causal effect*" OR  "causal discovery" OR  "causal machine learning" OR  "causal artificial intelligence" OR  "causal AI" OR  counterfactual* OR  "potential outcome*" OR  "structural causal model*" OR  "causal graph*" OR  "directed acyclic graph*" OR  DAG OR  "PC algorithm*" OR  "constraint-based causal discovery" OR  "target trial emulation" OR  "target trial" OR  "emulated trial" OR  "interrupted time series" OR  "regression discontinuity" OR  "regression discontinuity design*" OR  "instrumental variable*" OR  "instrumental variable analysis" OR  "synthetic control*" OR  "synthetic control method*" OR  "g-computation" OR  "g computation" OR  "g formula" OR  "g-formula" OR  "parametric g-formula" OR  "targeted maximum likelihood" OR  "targeted maximum likelihood estimation" OR  "targeted minimum loss" OR  "targeted learning" OR  TMLE OR  "doubly robust" OR  "doubly robust estimation" OR  "doubly robust estimator*" OR  "augmented inverse probability" OR  AIPW OR  "propensity score*" OR  "inverse probability weight*" OR  "inverse probability weighting" OR  "marginal structural model*" OR  "difference-in-differences" OR  "difference in differences" OR  "difference-in-difference" OR  "difference in difference" OR  "Bayesian structural time series" OR  CausalImpact OR  "causal impact" OR  "stepped wedge" OR  "quasi-experiment*" OR  "quasi experiment*" OR  "natural experiment*" OR  "treatment effect*" OR  "intervention effect*" OR  "average treatment effect*" OR  "conditional average treatment effect*" OR  "heterogeneous treatment effect*" OR  "individual treatment effect*" OR  "individualized treatment effect*" OR  "ATE estimation" OR  "CATE estimation" OR  "causal forest*" OR  "causal tree*" OR  "generalized random forest*" OR  "meta-learner*" OR  metalearner* OR  "S-learner*" OR  "T-learner*" OR  "X-learner*" OR  "R-learner*" OR  "S learner*" OR  "T learner*" OR  "X learner*" OR  "R learner*"  )  )  **AND**  (  TITLE-ABS-KEY(  "machine learning" OR  "machine learning model*" OR  "artificial intelligence" OR  "artificial intelligence model*" OR  "deep learning" OR  "neural network*" OR  "artificial neural network*" OR  "Bayesian network*" OR  "random forest*" OR  "gradient boosting" OR  XGBoost OR  transformer* OR  BERT OR  "large language model*" OR  LLM OR  LLMs OR  "generative artificial intelligence" OR  "generative AI" OR  ChatGPT OR  "natural language processing" OR  NLP OR  "predictive model*" OR  "prediction model*" OR  "clinical prediction model*" OR  "risk prediction" OR  "risk stratification" OR  "clinical decision support" OR  "clinical decision support system*" OR  "computerized clinical decision support" OR  "computerised clinical decision support" OR  "decision support" OR  CDSS OR  alert* OR  "automated alert*" OR  "clinical alert*" OR  "electronic alert*" OR  "algorithmic alert*" OR  "early warning" OR  "early warning score*" OR  "clinical deterioration" OR  "deterioration index" OR  "electronic health record algorithm*" OR  "EHR algorithm*"  )  )  **AND**  (  TITLE-ABS-KEY(  nurs* OR  midwif* OR  "nursing care" OR  "nursing workflow" OR  "nursing documentation" OR  "nursing intervention*" OR  "nurse-led" OR  "nurse led" OR  "nurse-facing" OR  "nurse facing" OR  "nurse-implemented" OR  "nurse implemented" OR  "nurse alert*" OR  "nursing alert*" OR  "nurse response" OR  "nurse responses" OR  "nursing response" OR  "nursing responses" OR  "care delivery" OR  "care workflow" OR  "clinical workflow" OR  "patient monitoring" OR  triage OR  "escalation of care" OR  bedside OR  ward OR  wards OR  inpatient* OR  "health personnel" OR  "allied health personnel"  )  )  AND PUBYEAR > 2014  AND PUBYEAR < 2027 | | |
| **Embase (Elsevier)** | Search date: 11 June 2026 Publication years: 2015-2026 Fields: title, abstract, and keywords (ti, ab, kw) Language filter: none | **1,185** |
| **Complete search strategy**  (  'causal inference':ti,ab,kw OR  'causal effect*':ti,ab,kw OR  'causal discovery':ti,ab,kw OR  'causal machine learning':ti,ab,kw OR  'causal artificial intelligence':ti,ab,kw OR  'causal AI':ti,ab,kw OR  counterfactual*:ti,ab,kw OR  'potential outcome*':ti,ab,kw OR  'structural causal model*':ti,ab,kw OR  'causal graph*':ti,ab,kw OR  'directed acyclic graph*':ti,ab,kw OR  DAG:ti,ab,kw OR  'PC algorithm*':ti,ab,kw OR  'constraint-based causal discovery':ti,ab,kw OR  'target trial emulation':ti,ab,kw OR  'target trial':ti,ab,kw OR  'emulated trial':ti,ab,kw OR  'interrupted time series':ti,ab,kw OR  'regression discontinuity':ti,ab,kw OR  'regression discontinuity design*':ti,ab,kw OR  'instrumental variable*':ti,ab,kw OR  'instrumental variable analysis':ti,ab,kw OR  'synthetic control*':ti,ab,kw OR  'synthetic control method*':ti,ab,kw OR  'g-computation':ti,ab,kw OR  'g computation':ti,ab,kw OR  'g formula':ti,ab,kw OR  'g-formula':ti,ab,kw OR  'parametric g-formula':ti,ab,kw OR  'targeted maximum likelihood':ti,ab,kw OR  'targeted maximum likelihood estimation':ti,ab,kw OR  'targeted minimum loss':ti,ab,kw OR  'targeted learning':ti,ab,kw OR  TMLE:ti,ab,kw OR  'doubly robust':ti,ab,kw OR  'doubly robust estimation':ti,ab,kw OR  'doubly robust estimator*':ti,ab,kw OR  'augmented inverse probability':ti,ab,kw OR  AIPW:ti,ab,kw OR  'propensity score*':ti,ab,kw OR  'inverse probability weight*':ti,ab,kw OR  'inverse probability weighting':ti,ab,kw OR  'marginal structural model*':ti,ab,kw OR  'difference-in-differences':ti,ab,kw OR  'difference in differences':ti,ab,kw OR  'difference-in-difference':ti,ab,kw OR  'difference in difference':ti,ab,kw OR  'Bayesian structural time series':ti,ab,kw OR  CausalImpact:ti,ab,kw OR  'causal impact':ti,ab,kw OR  'stepped wedge':ti,ab,kw OR  'quasi-experiment*':ti,ab,kw OR  'quasi experiment*':ti,ab,kw OR  'natural experiment*':ti,ab,kw OR  'treatment effect*':ti,ab,kw OR  'intervention effect*':ti,ab,kw OR  'average treatment effect*':ti,ab,kw OR  'conditional average treatment effect*':ti,ab,kw OR  'heterogeneous treatment effect*':ti,ab,kw OR  'individual treatment effect*':ti,ab,kw OR  'individualized treatment effect*':ti,ab,kw OR  'ATE estimation':ti,ab,kw OR  'CATE estimation':ti,ab,kw OR  'causal forest*':ti,ab,kw OR  'causal tree*':ti,ab,kw OR  'generalized random forest*':ti,ab,kw OR  'meta-learner*':ti,ab,kw OR  'meta learner*':ti,ab,kw OR  metalearner*:ti,ab,kw OR  'S-learner*':ti,ab,kw OR  'T-learner*':ti,ab,kw OR  'X-learner*':ti,ab,kw OR  'R-learner*':ti,ab,kw OR  'S learner*':ti,ab,kw OR  'T learner*':ti,ab,kw OR  'X learner*':ti,ab,kw OR  'R learner*':ti,ab,kw  )  **AND**  (  'machine learning':ti,ab,kw OR  'machine learning model*':ti,ab,kw OR  'artificial intelligence':ti,ab,kw OR  'artificial intelligence model*':ti,ab,kw OR  'deep learning':ti,ab,kw OR  'neural network*':ti,ab,kw OR  'artificial neural network*':ti,ab,kw OR  'Bayesian network*':ti,ab,kw OR  'random forest*':ti,ab,kw OR  'gradient boosting':ti,ab,kw OR  XGBoost:ti,ab,kw OR  transformer*:ti,ab,kw OR  BERT:ti,ab,kw OR  'large language model*':ti,ab,kw OR  LLM:ti,ab,kw OR  LLMs:ti,ab,kw OR  'generative artificial intelligence':ti,ab,kw OR  'generative AI':ti,ab,kw OR  ChatGPT:ti,ab,kw OR  'natural language processing':ti,ab,kw OR  NLP:ti,ab,kw OR  'predictive model*':ti,ab,kw OR  'prediction model*':ti,ab,kw OR  'clinical prediction model*':ti,ab,kw OR  'risk prediction':ti,ab,kw OR  'risk stratification':ti,ab,kw OR  'clinical decision support':ti,ab,kw OR  'clinical decision support system*':ti,ab,kw OR  'computerized clinical decision support':ti,ab,kw OR  'computerised clinical decision support':ti,ab,kw OR  'decision support':ti,ab,kw OR  CDSS:ti,ab,kw OR  alert*:ti,ab,kw OR  'automated alert*':ti,ab,kw OR  'clinical alert*':ti,ab,kw OR  'electronic alert*':ti,ab,kw OR  'algorithmic alert*':ti,ab,kw OR  'early warning':ti,ab,kw OR  'early warning score*':ti,ab,kw OR  'clinical deterioration':ti,ab,kw OR  'deterioration index':ti,ab,kw OR  'electronic health record algorithm*':ti,ab,kw OR  'EHR algorithm*':ti,ab,kw  )  **AND**  (  nurs*:ti,ab,kw OR  midwif*:ti,ab,kw OR  'nursing care':ti,ab,kw OR  'nursing workflow':ti,ab,kw OR  'nursing documentation':ti,ab,kw OR  'nursing intervention*':ti,ab,kw OR  'nurse-led':ti,ab,kw OR  'nurse led':ti,ab,kw OR  'nurse-facing':ti,ab,kw OR  'nurse facing':ti,ab,kw OR  'nurse-implemented':ti,ab,kw OR  'nurse implemented':ti,ab,kw OR  'nurse alert*':ti,ab,kw OR  'nursing alert*':ti,ab,kw OR  'nurse response':ti,ab,kw OR  'nurse responses':ti,ab,kw OR  'nursing response':ti,ab,kw OR  'nursing responses':ti,ab,kw OR  'care delivery':ti,ab,kw OR  'care workflow':ti,ab,kw OR  'clinical workflow':ti,ab,kw OR  'patient monitoring':ti,ab,kw OR  triage:ti,ab,kw OR  'escalation of care':ti,ab,kw OR  bedside:ti,ab,kw OR  ward:ti,ab,kw OR  wards:ti,ab,kw OR  inpatient*:ti,ab,kw OR  'health personnel':ti,ab,kw OR  'allied health personnel':ti,ab,kw  )  AND [2015-2026]/py | | |
| **CINAHL (EBSCOhost)** | Search date: 11 June 2026 Date range: 1 January 2015-11 June 2026 Fields: title, abstract, and nursing/midwifery CINAHL subject headings Language filter: none | **164** |
| **Complete search strategy**  **S1a. Core causal and quasi-experimental terms**  TI (  "causal inference" OR "causal effect*" OR "causal discovery" OR  counterfactual* OR "potential outcome*" OR "structural causal model*" OR  "causal graph*" OR "directed acyclic graph*" OR DAG OR  "PC algorithm*" OR "constraint-based causal discovery" OR  "target trial emulation" OR "target trial" OR "emulated trial" OR  "interrupted time series" OR "regression discontinuity" OR  "regression discontinuity design*" OR "instrumental variable*" OR  "instrumental variable analysis" OR "synthetic control*" OR  "synthetic control method*" OR "g-computation" OR "g computation" OR  "g formula" OR "g-formula" OR "parametric g-formula" OR  "targeted maximum likelihood" OR "targeted maximum likelihood estimation" OR  "targeted minimum loss" OR "targeted learning" OR TMLE OR  "doubly robust" OR "doubly robust estimation" OR "doubly robust estimator*" OR  "augmented inverse probability" OR AIPW OR "propensity score*" OR  "inverse probability weight*" OR "inverse probability weighting" OR  "marginal structural model*" OR "difference-in-differences" OR  "difference in differences" OR "difference-in-difference" OR  "difference in difference" OR "Bayesian structural time series" OR  CausalImpact OR "causal impact" OR "stepped wedge" OR  "quasi-experiment*" OR "quasi experiment*" OR "natural experiment*" OR  "treatment effect*" OR "intervention effect*" OR  "average treatment effect*" OR "conditional average treatment effect*" OR  "heterogeneous treatment effect*" OR "individual treatment effect*" OR  "individualized treatment effect*" OR "ATE estimation" OR "CATE estimation"  )  OR  AB (  "causal inference" OR "causal effect*" OR "causal discovery" OR  counterfactual* OR "potential outcome*" OR "structural causal model*" OR  "causal graph*" OR "directed acyclic graph*" OR DAG OR  "PC algorithm*" OR "constraint-based causal discovery" OR  "target trial emulation" OR "target trial" OR "emulated trial" OR  "interrupted time series" OR "regression discontinuity" OR  "regression discontinuity design*" OR "instrumental variable*" OR  "instrumental variable analysis" OR "synthetic control*" OR  "synthetic control method*" OR "g-computation" OR "g computation" OR  "g formula" OR "g-formula" OR "parametric g-formula" OR  "targeted maximum likelihood" OR "targeted maximum likelihood estimation" OR  "targeted minimum loss" OR "targeted learning" OR TMLE OR  "doubly robust" OR "doubly robust estimation" OR "doubly robust estimator*" OR  "augmented inverse probability" OR AIPW OR "propensity score*" OR  "inverse probability weight*" OR "inverse probability weighting" OR  "marginal structural model*" OR "difference-in-differences" OR  "difference in differences" OR "difference-in-difference" OR  "difference in difference" OR "Bayesian structural time series" OR  CausalImpact OR "causal impact" OR "stepped wedge" OR  "quasi-experiment*" OR "quasi experiment*" OR "natural experiment*" OR  "treatment effect*" OR "intervention effect*" OR  "average treatment effect*" OR "conditional average treatment effect*" OR  "heterogeneous treatment effect*" OR "individual treatment effect*" OR  "individualized treatment effect*" OR "ATE estimation" OR "CATE estimation"  )  **S1b. Causal machine-learning terms**  TI (  "causal machine learning" OR "causal artificial intelligence" OR "causal AI" OR  "causal forest*" OR "causal tree*" OR "generalized random forest*" OR  "meta-learner*" OR metalearner* OR "S-learner*" OR "T-learner*" OR  "X-learner*" OR "R-learner*" OR "S learner*" OR "T learner*" OR  "X learner*" OR "R learner*"  )  OR  AB (  "causal machine learning" OR "causal artificial intelligence" OR "causal AI" OR  "causal forest*" OR "causal tree*" OR "generalized random forest*" OR  "meta-learner*" OR metalearner* OR "S-learner*" OR "T-learner*" OR  "X-learner*" OR "R-learner*" OR "S learner*" OR "T learner*" OR  "X learner*" OR "R learner*"  )  **S1. Final causal block**  **S1a OR S1b = 78,996**  **S2a. AI and ML terms**  TI (  "machine learning" OR "machine learning model*" OR  "artificial intelligence" OR "artificial intelligence model*" OR  "deep learning" OR "neural network*" OR "artificial neural network*" OR  "Bayesian network*" OR "random forest*" OR "gradient boosting" OR  XGBoost OR transformer* OR BERT OR "large language model*" OR  LLM OR LLMs OR "generative artificial intelligence" OR "generative AI" OR  ChatGPT OR "natural language processing" OR NLP OR  "predictive model*" OR "prediction model*" OR "clinical prediction model*" OR  "risk prediction" OR "risk stratification"  )  OR  AB (  "machine learning" OR "machine learning model*" OR  "artificial intelligence" OR "artificial intelligence model*" OR  "deep learning" OR "neural network*" OR "artificial neural network*" OR  "Bayesian network*" OR "random forest*" OR "gradient boosting" OR  XGBoost OR transformer* OR BERT OR "large language model*" OR  LLM OR LLMs OR "generative artificial intelligence" OR "generative AI" OR  ChatGPT OR "natural language processing" OR NLP OR  "predictive model*" OR "prediction model*" OR "clinical prediction model*" OR  "risk prediction" OR "risk stratification"  )  **S2b. Clinical decision-support, alert, and workflow terms**  TI (  "clinical decision support" OR "clinical decision support system*" OR  "computerized clinical decision support" OR "computerised clinical decision support" OR  "decision support" OR CDSS OR alert* OR "automated alert*" OR  "clinical alert*" OR "electronic alert*" OR "algorithmic alert*" OR  "early warning" OR "early warning score*" OR "clinical deterioration" OR  "deterioration index" OR "electronic health record algorithm*" OR  "EHR algorithm*"  )  OR  AB (  "clinical decision support" OR "clinical decision support system*" OR  "computerized clinical decision support" OR "computerised clinical decision support" OR  "decision support" OR CDSS OR alert* OR "automated alert*" OR  "clinical alert*" OR "electronic alert*" OR "algorithmic alert*" OR  "early warning" OR "early warning score*" OR "clinical deterioration" OR  "deterioration index" OR "electronic health record algorithm*" OR  "EHR algorithm*"  )  **S2. Final AI/ML/clinical decision-support block**  **S2a OR S2b = 128,095**  **S3. Nursing, midwifery, and nursing-relevant workflow block = 1,011,229**  TI (  nurs* OR midwif* OR  "nursing care" OR "nursing workflow" OR "nursing documentation" OR  "nursing intervention*" OR "nurse-led" OR "nurse led" OR  "nurse-facing" OR "nurse facing" OR "nurse-implemented" OR  "nurse implemented" OR "nurse alert*" OR "nursing alert*" OR  "nurse response" OR "nurse responses" OR "nursing response" OR  "nursing responses" OR "care delivery" OR "care workflow" OR  "clinical workflow" OR "patient monitoring" OR triage OR  "escalation of care" OR bedside OR ward OR wards OR inpatient* OR  "health personnel" OR "allied health personnel"  )  OR  AB (  nurs* OR midwif* OR  "nursing care" OR "nursing workflow" OR "nursing documentation" OR  "nursing intervention*" OR "nurse-led" OR "nurse led" OR  "nurse-facing" OR "nurse facing" OR "nurse-implemented" OR  "nurse implemented" OR "nurse alert*" OR "nursing alert*" OR  "nurse response" OR "nurse responses" OR "nursing response" OR  "nursing responses" OR "care delivery" OR "care workflow" OR  "clinical workflow" OR "patient monitoring" OR triage OR  "escalation of care" OR bedside OR ward OR wards OR inpatient* OR  "health personnel" OR "allied health personnel"  )  OR  MH "Nursing+"  OR  MH "Nurses+"  OR  MH "Nursing Care+"  OR  MH "Nursing Staff+"  OR  MH "Nurse Practitioners+"  OR  MH "Nursing Informatics+"  OR  MH "Midwifery+"  **S4. Final combination**  **S1 AND S2 AND S3 = 232**  **Limited to 1 January 2015-11 June 2026 = 164** | | |
| **Web of Science Core Collection** | Search date: 11 June 2026 Publication years: 2015-2026 Field: Topic (title, abstract, author keywords, and Keywords Plus) Language filter: none | **826** |
| **Complete search strategy**  **#1. Causal and causally oriented methods**  TS=(  "causal inference" OR  "causal effect*" OR  "causal discovery" OR  "causal machine learning" OR  "causal artificial intelligence" OR  "causal AI" OR  counterfactual* OR  "potential outcome*" OR  "structural causal model*" OR  "causal graph*" OR  "directed acyclic graph*" OR  DAG OR  "PC algorithm*" OR  "constraint-based causal discovery" OR  "target trial emulation" OR  "target trial" OR  "emulated trial" OR  "interrupted time series" OR  "regression discontinuity" OR  "regression discontinuity design*" OR  "instrumental variable*" OR  "instrumental variable analysis" OR  "synthetic control*" OR  "synthetic control method*" OR  "g-computation" OR  "g computation" OR  "g formula" OR  "g-formula" OR  "parametric g-formula" OR  "targeted maximum likelihood" OR  "targeted maximum likelihood estimation" OR  "targeted minimum loss" OR  "targeted learning" OR  TMLE OR  "doubly robust" OR  "doubly robust estimation" OR  "doubly robust estimator*" OR  "augmented inverse probability" OR  AIPW OR  "propensity score*" OR  "inverse probability weight*" OR  "inverse probability weighting" OR  "marginal structural model*" OR  "difference-in-differences" OR  "difference in differences" OR  "difference-in-difference" OR  "difference in difference" OR  "Bayesian structural time series" OR  CausalImpact OR  "causal impact" OR  "stepped wedge" OR  "quasi-experiment*" OR  "quasi experiment*" OR  "natural experiment*" OR  "treatment effect*" OR  "intervention effect*" OR  "average treatment effect*" OR  "conditional average treatment effect*" OR  "heterogeneous treatment effect*" OR  "individual treatment effect*" OR  "individualized treatment effect*" OR  "ATE estimation" OR  "CATE estimation" OR  "causal forest*" OR  "causal tree*" OR  "generalized random forest*" OR  "meta-learner*" OR  "meta learner*" OR  metalearner* OR  "metalearner*" OR  "S-learner*" OR  "T-learner*" OR  "X-learner*" OR  "R-learner*" OR  "S learner*" OR  "T learner*" OR  "X learner*" OR  "R learner*"  )  **#2. AI, ML, and clinical decision-support terms**  TS=(  "machine learning" OR  "machine learning model*" OR  "artificial intelligence" OR  "artificial intelligence model*" OR  "deep learning" OR  "neural network*" OR  "artificial neural network*" OR  "Bayesian network*" OR  "random forest*" OR  "gradient boosting" OR  XGBoost OR  transformer* OR  BERT OR  "large language model*" OR  LLM OR  LLMs OR  "generative artificial intelligence" OR  "generative AI" OR  ChatGPT OR  "natural language processing" OR  NLP OR  "predictive model*" OR  "prediction model*" OR  "clinical prediction model*" OR  "risk prediction" OR  "risk stratification" OR  "clinical decision support" OR  "clinical decision support system*" OR  "computerized clinical decision support" OR  "computerised clinical decision support" OR  "decision support" OR  CDSS OR  alert* OR  "automated alert*" OR  "clinical alert*" OR  "electronic alert*" OR  "algorithmic alert*" OR  "early warning" OR  "early warning score*" OR  "clinical deterioration" OR  "deterioration index" OR  "electronic health record algorithm*" OR  "EHR algorithm*"  )  **#3. Nursing, midwifery, and nursing-relevant workflow terms**  TS=(  nurs* OR  midwif* OR  "nursing care" OR  "nursing workflow" OR  "nursing documentation" OR  "nursing intervention*" OR  "nurse-led" OR  "nurse led" OR  "nurse-facing" OR  "nurse facing" OR  "nurse-implemented" OR  "nurse implemented" OR  "nurse alert*" OR  "nursing alert*" OR  "nurse response" OR  "nurse responses" OR  "nursing response" OR  "nursing responses" OR  "care delivery" OR  "care workflow" OR  "clinical workflow" OR  "patient monitoring" OR  triage OR  "escalation of care" OR  bedside OR  ward OR  wards OR  inpatient* OR  "health personnel" OR  "allied health personnel"  )  **#4. Final combination**  **#1 AND #2 AND #3** | | |
| **IEEE Xplore** | Search date: 11 June 2026 Publication years: 2015-2026 Search field: All Metadata Content types: conferences and journals Language filter: none | **65** |
| **Complete search strategy**  (  "causal inference" OR  "causal effect" OR "causal effects" OR  "causal discovery" OR  "causal machine learning" OR  "causal artificial intelligence" OR  "causal AI" OR  counterfactual OR counterfactuals OR  "potential outcome" OR "potential outcomes" OR  "structural causal model" OR "structural causal models" OR  "causal graph" OR "causal graphs" OR  "directed acyclic graph" OR "directed acyclic graphs" OR  DAG OR  "PC algorithm" OR "PC algorithms" OR  "constraint-based causal discovery" OR  "target trial emulation" OR  "target trial" OR  "target trials" OR  "emulated trial" OR  "emulated trials" OR  "interrupted time series" OR  "regression discontinuity" OR  "regression discontinuity design" OR  "regression discontinuity designs" OR  "instrumental variable" OR  "instrumental variables" OR  "instrumental variable analysis" OR  "synthetic control" OR  "synthetic controls" OR  "synthetic control method" OR  "synthetic control methods" OR  "g-computation" OR  "g computation" OR  "g formula" OR  "g-formula" OR  "parametric g-formula" OR  "targeted maximum likelihood" OR  "targeted maximum likelihood estimation" OR  "targeted minimum loss" OR  "targeted learning" OR  TMLE OR  "doubly robust" OR  "doubly robust estimation" OR  "doubly robust estimator" OR  "doubly robust estimators" OR  "augmented inverse probability" OR  AIPW OR  "propensity score" OR  "propensity scores" OR  "inverse probability weighting" OR  "inverse probability weight" OR  "inverse probability weights" OR  "marginal structural model" OR  "marginal structural models" OR  "difference-in-differences" OR  "difference in differences" OR  "difference-in-difference" OR  "difference in difference" OR  "Bayesian structural time series" OR  CausalImpact OR  "causal impact" OR  "stepped wedge" OR  "quasi-experiment" OR  "quasi-experiments" OR  "quasi-experimental" OR  "quasi experiment" OR  "quasi experiments" OR  "quasi experimental" OR  "natural experiment" OR  "natural experiments" OR  "treatment effect" OR  "treatment effects" OR  "intervention effect" OR  "intervention effects" OR  "average treatment effect" OR  "average treatment effects" OR  "conditional average treatment effect" OR  "conditional average treatment effects" OR  "heterogeneous treatment effect" OR  "heterogeneous treatment effects" OR  "individual treatment effect" OR  "individual treatment effects" OR  "individualized treatment effect" OR  "individualized treatment effects" OR  "ATE estimation" OR  "CATE estimation" OR  "causal forest" OR  "causal forests" OR  "causal tree" OR  "causal trees" OR  "generalized random forest" OR  "generalized random forests" OR  "meta-learner" OR  "meta-learners" OR  metalearner OR  metalearners OR  "S-learner" OR "S-learners" OR  "T-learner" OR "T-learners" OR  "X-learner" OR "X-learners" OR  "R-learner" OR "R-learners" OR  "S learner" OR "S learners" OR  "T learner" OR "T learners" OR  "X learner" OR "X learners" OR  "R learner" OR "R learners"  )  **AND**  (  "machine learning" OR  "machine learning model" OR  "machine learning models" OR  "artificial intelligence" OR  "artificial intelligence model" OR  "artificial intelligence models" OR  "deep learning" OR  "neural network" OR  "neural networks" OR  "artificial neural network" OR  "artificial neural networks" OR  "Bayesian network" OR  "Bayesian networks" OR  "random forest" OR  "random forests" OR  "gradient boosting" OR  XGBoost OR  transformer OR transformers OR  BERT OR  "large language model" OR  "large language models" OR  LLM OR LLMs OR  "generative artificial intelligence" OR  "generative AI" OR  ChatGPT OR  "natural language processing" OR  NLP OR  "predictive model" OR  "predictive models" OR  "prediction model" OR  "prediction models" OR  "clinical prediction model" OR  "clinical prediction models" OR  "risk prediction" OR  "risk stratification" OR  "clinical decision support" OR  "clinical decision support system" OR  "clinical decision support systems" OR  "computerized clinical decision support" OR  "computerised clinical decision support" OR  "decision support" OR  CDSS OR  alert OR alerts OR  "automated alert" OR  "automated alerts" OR  "clinical alert" OR  "clinical alerts" OR  "electronic alert" OR  "electronic alerts" OR  "algorithmic alert" OR  "algorithmic alerts" OR  "early warning" OR  "early warning score" OR  "early warning scores" OR  "clinical deterioration" OR  "deterioration index" OR  "electronic health record algorithm" OR  "electronic health record algorithms" OR  "EHR algorithm" OR  "EHR algorithms"  )  **AND**  (  nursing OR  nurse OR  nurses OR  midwife OR  midwives OR  midwifery OR  "nursing care" OR  "nursing workflow" OR  "nursing documentation" OR  "nursing intervention" OR  "nursing interventions" OR  "nurse-led" OR  "nurse led" OR  "nurse-facing" OR  "nurse facing" OR  "nurse-implemented" OR  "nurse implemented" OR  "nurse alert" OR  "nurse alerts" OR  "nursing alert" OR  "nursing alerts" OR  "nurse response" OR  "nurse responses" OR  "nursing response" OR  "nursing responses" OR  "care delivery" OR  "care workflow" OR  "clinical workflow" OR  "patient monitoring" OR  triage OR  "escalation of care" OR  bedside OR  ward OR  wards OR  inpatient OR  inpatients OR  "health personnel" OR  "allied health personnel"  ) | | |
| **ACM Digital Library (The ACM Guide to Computing Literature)** | Search date: 11 June 2026 Date range: 1 January 2015-11 June 2026 Fields: title or abstract The three blocks were executed as one expression combined with AND Language filter: none | **19** |
| **Complete search strategy**  **Block 1. Causal and causally oriented terms**  (  Title:(  "causal inference" OR  "causal effect*" OR  "causal discovery" OR  "causal machine learning" OR  "causal artificial intelligence" OR  "causal AI" OR  counterfactual* OR  "potential outcome*" OR  "structural causal model*" OR  "causal graph*" OR  "directed acyclic graph*" OR  DAG OR  "PC algorithm*" OR  "constraint-based causal discovery" OR  "target trial emulation" OR  "target trial" OR  "emulated trial" OR  "interrupted time series" OR  "regression discontinuity" OR  "regression discontinuity design*" OR  "instrumental variable*" OR  "instrumental variable analysis" OR  "synthetic control*" OR  "synthetic control method*" OR  "g-computation" OR  "g computation" OR  "g formula" OR  "g-formula" OR  "parametric g-formula" OR  "targeted maximum likelihood" OR  "targeted maximum likelihood estimation" OR  "targeted minimum loss" OR  "targeted learning" OR  TMLE OR  "doubly robust" OR  "doubly robust estimation" OR  "doubly robust estimator*" OR  "augmented inverse probability" OR  AIPW OR  "propensity score*" OR  "inverse probability weight*" OR  "inverse probability weighting" OR  "marginal structural model*" OR  "difference-in-differences" OR  "difference in differences" OR  "difference-in-difference" OR  "difference in difference" OR  "Bayesian structural time series" OR  CausalImpact OR  "causal impact" OR  "stepped wedge" OR  "quasi-experiment*" OR  "quasi experiment*" OR  "natural experiment*" OR  "treatment effect*" OR  "intervention effect*" OR  "average treatment effect*" OR  "conditional average treatment effect*" OR  "heterogeneous treatment effect*" OR  "individual treatment effect*" OR  "individualized treatment effect*" OR  "ATE estimation" OR  "CATE estimation" OR  "causal forest*" OR  "causal tree*" OR  "generalized random forest*" OR  "meta-learner*" OR  "meta learner*" OR  metalearner* OR  "S-learner*" OR  "T-learner*" OR  "X-learner*" OR  "R-learner*" OR  "S learner*" OR  "T learner*" OR  "X learner*" OR  "R learner*"  )  OR  Abstract:(  "causal inference" OR  "causal effect*" OR  "causal discovery" OR  "causal machine learning" OR  "causal artificial intelligence" OR  "causal AI" OR  counterfactual* OR  "potential outcome*" OR  "structural causal model*" OR  "causal graph*" OR  "directed acyclic graph*" OR  DAG OR  "PC algorithm*" OR  "constraint-based causal discovery" OR  "target trial emulation" OR  "target trial" OR  "emulated trial" OR  "interrupted time series" OR  "regression discontinuity" OR  "regression discontinuity design*" OR  "instrumental variable*" OR  "instrumental variable analysis" OR  "synthetic control*" OR  "synthetic control method*" OR  "g-computation" OR  "g computation" OR  "g formula" OR  "g-formula" OR  "parametric g-formula" OR  "targeted maximum likelihood" OR  "targeted maximum likelihood estimation" OR  "targeted minimum loss" OR  "targeted learning" OR  TMLE OR  "doubly robust" OR  "doubly robust estimation" OR  "doubly robust estimator*" OR  "augmented inverse probability" OR  AIPW OR  "propensity score*" OR  "inverse probability weight*" OR  "inverse probability weighting" OR  "marginal structural model*" OR  "difference-in-differences" OR  "difference in differences" OR  "difference-in-difference" OR  "difference in difference" OR  "Bayesian structural time series" OR  CausalImpact OR  "causal impact" OR  "stepped wedge" OR  "quasi-experiment*" OR  "quasi experiment*" OR  "natural experiment*" OR  "treatment effect*" OR  "intervention effect*" OR  "average treatment effect*" OR  "conditional average treatment effect*" OR  "heterogeneous treatment effect*" OR  "individual treatment effect*" OR  "individualized treatment effect*" OR  "ATE estimation" OR  "CATE estimation" OR  "causal forest*" OR  "causal tree*" OR  "generalized random forest*" OR  "meta-learner*" OR  "meta learner*" OR  metalearner* OR  "S-learner*" OR  "T-learner*" OR  "X-learner*" OR  "R-learner*" OR  "S learner*" OR  "T learner*" OR  "X learner*" OR  "R learner*")  )  **AND**  **Block 2. AI, ML, and clinical decision-support terms**  (  Title:(  "machine learning" OR  "machine learning model*" OR  "artificial intelligence" OR  "artificial intelligence model*" OR  "deep learning" OR  "neural network*" OR  "artificial neural network*" OR  "Bayesian network*" OR  "random forest*" OR  "gradient boosting" OR  XGBoost OR  transformer* OR  BERT OR  "large language model*" OR  LLM OR  LLMs OR  "generative artificial intelligence" OR  "generative AI" OR  ChatGPT OR  "natural language processing" OR  NLP OR  "predictive model*" OR  "prediction model*" OR  "clinical prediction model*" OR  "risk prediction" OR  "risk stratification" OR  "clinical decision support" OR  "clinical decision support system*" OR  "computerized clinical decision support" OR  "computerised clinical decision support" OR  "decision support" OR  CDSS OR  alert* OR  "automated alert*" OR  "clinical alert*" OR  "electronic alert*" OR  "algorithmic alert*" OR  "early warning" OR  "early warning score*" OR  "clinical deterioration" OR  "deterioration index" OR  "electronic health record algorithm*" OR  "EHR algorithm*"  )  OR  Abstract:(  "machine learning" OR  "machine learning model*" OR  "artificial intelligence" OR  "artificial intelligence model*" OR  "deep learning" OR  "neural network*" OR  "artificial neural network*" OR  "Bayesian network*" OR  "random forest*" OR  "gradient boosting" OR  XGBoost OR  transformer* OR  BERT OR  "large language model*" OR  LLM OR  LLMs OR  "generative artificial intelligence" OR  "generative AI" OR  ChatGPT OR  "natural language processing" OR  NLP OR  "predictive model*" OR  "prediction model*" OR  "clinical prediction model*" OR  "risk prediction" OR  "risk stratification" OR  "clinical decision support" OR  "clinical decision support system*" OR  "computerized clinical decision support" OR  "computerised clinical decision support" OR  "decision support" OR  CDSS OR  alert* OR  "automated alert*" OR  "clinical alert*" OR  "electronic alert*" OR  "algorithmic alert*" OR  "early warning" OR  "early warning score*" OR  "clinical deterioration" OR  "deterioration index" OR  "electronic health record algorithm*" OR  "EHR algorithm*")  )  **AND**  **Block 3. Nursing, midwifery, and nursing-relevant workflow terms**  (  Title:(  nurs* OR  midwif* OR  "nursing care" OR  "nursing workflow" OR  "nursing documentation" OR  "nursing intervention*" OR  "nurse-led" OR  "nurse led" OR  "nurse-facing" OR  "nurse facing" OR  "nurse-implemented" OR  "nurse implemented" OR  "nurse alert*" OR  "nursing alert*" OR  "nurse response" OR  "nurse responses" OR  "nursing response" OR  "nursing responses" OR  "care delivery" OR  "care workflow" OR  "clinical workflow" OR  "patient monitoring" OR  triage OR  "escalation of care" OR  bedside OR  ward OR  wards OR  inpatient* OR  "health personnel" OR  "allied health personnel"  )  OR  Abstract:(  nurs* OR  midwif* OR  "nursing care" OR  "nursing workflow" OR  "nursing documentation" OR  "nursing intervention*" OR  "nurse-led" OR  "nurse led" OR  "nurse-facing" OR  "nurse facing" OR  "nurse-implemented" OR  "nurse implemented" OR  "nurse alert*" OR  "nursing alert*" OR  "nurse response" OR  "nurse responses" OR  "nursing response" OR  "nursing responses" OR  "care delivery" OR  "care workflow" OR  "clinical workflow" OR  "patient monitoring" OR  triage OR  "escalation of care" OR  bedside OR  ward OR  wards OR  inpatient* OR  "health personnel" OR  "allied health personnel")  ) | | |
| **ProQuest Dissertations & Theses Global** | Search date: 11 June 2026 Date range: 1 January 2015-11 June 2026 Search field: Anywhere except full text (NOFT) Language filter: none Full-text filter: none | **426** |
| **Complete search strategy**  NOFT(  (  "causal inference" OR  "causal effect*" OR  "causal discovery" OR  "causal machine learning" OR  "causal artificial intelligence" OR  "causal AI" OR  counterfactual* OR  "potential outcome*" OR  "structural causal model*" OR  "causal graph*" OR  "directed acyclic graph*" OR  DAG OR  "PC algorithm*" OR  "constraint-based causal discovery" OR  "target trial emulation" OR  "target trial" OR  "emulated trial" OR  "interrupted time series" OR  "regression discontinuity" OR  "regression discontinuity design*" OR  "instrumental variable*" OR  "instrumental variable analysis" OR  "synthetic control*" OR  "synthetic control method*" OR  "g-computation" OR  "g computation" OR  "g formula" OR  "g-formula" OR  "parametric g-formula" OR  "targeted maximum likelihood" OR  "targeted maximum likelihood estimation" OR  "targeted minimum loss" OR  "targeted learning" OR  TMLE OR  "doubly robust" OR  "doubly robust estimation" OR  "doubly robust estimator*" OR  "augmented inverse probability" OR  AIPW OR  "propensity score*" OR  "inverse probability weight*" OR  "inverse probability weighting" OR  "marginal structural model*" OR  "difference-in-differences" OR  "difference in differences" OR  "difference-in-difference" OR  "difference in difference" OR  "Bayesian structural time series" OR  CausalImpact OR  "causal impact" OR  "stepped wedge" OR  "quasi-experiment*" OR  "quasi experiment*" OR  "natural experiment*" OR  "treatment effect*" OR  "intervention effect*" OR  "average treatment effect*" OR  "conditional average treatment effect*" OR  "heterogeneous treatment effect*" OR  "individual treatment effect*" OR  "individualized treatment effect*" OR  "ATE estimation" OR  "CATE estimation" OR  "causal forest*" OR  "causal tree*" OR  "generalized random forest*" OR  "meta-learner*" OR  "meta learner*" OR  metalearner* OR  "S-learner*" OR  "T-learner*" OR  "X-learner*" OR  "R-learner*" OR  "S learner*" OR  "T learner*" OR  "X learner*" OR  "R learner*"  )  **AND**  (  "machine learning" OR  "machine learning model*" OR  "artificial intelligence" OR  "artificial intelligence model*" OR  "deep learning" OR  "neural network*" OR  "artificial neural network*" OR  "Bayesian network*" OR  "random forest*" OR  "gradient boosting" OR  XGBoost OR  transformer* OR  BERT OR  "large language model*" OR  LLM OR  LLMs OR  "generative artificial intelligence" OR  "generative AI" OR  ChatGPT OR  "natural language processing" OR  NLP OR  "predictive model*" OR  "prediction model*" OR  "clinical prediction model*" OR  "risk prediction" OR  "risk stratification" OR  "clinical decision support" OR  "clinical decision support system*" OR  "computerized clinical decision support" OR  "computerised clinical decision support" OR  "decision support" OR  CDSS OR  alert* OR  "automated alert*" OR  "clinical alert*" OR  "electronic alert*" OR  "algorithmic alert*" OR  "early warning" OR  "early warning score*" OR  "clinical deterioration" OR  "deterioration index" OR  "electronic health record algorithm*" OR  "EHR algorithm*"  )  **AND**  (  nurs* OR  midwif* OR  "nursing care" OR  "nursing workflow" OR  "nursing documentation" OR  "nursing intervention*" OR  "nurse-led" OR  "nurse led" OR  "nurse-facing" OR  "nurse facing" OR  "nurse-implemented" OR  "nurse implemented" OR  "nurse alert*" OR  "nursing alert*" OR  "nurse response" OR  "nurse responses" OR  "nursing response" OR  "nursing responses" OR  "care delivery" OR  "care workflow" OR  "clinical workflow" OR  "patient monitoring" OR  triage OR  "escalation of care" OR  bedside OR  ward OR  wards OR  inpatient* OR  "health personnel" OR  "allied health personnel"  )  ) | | |
| ***Note***. All searches were conducted on 11 June 2026. No language filter was applied at the search stage. Searches covered records published from 1 January 2015 to 11 June 2026. The eligibility restriction to English-language reports was applied at title and abstract screening. The searches yielded 4,918 records before deduplication. | | |
