# Supplementary material for: Machine Learning in Causal Effect Estimation and Causal Evaluation of ML‐Enabled Interventions in Nursing‐Related Research: A Scoping Review
Source: Nurs Health Sci. 2026 Oct 7;28(4):e70431. doi: 10.1111/nhs.70431 (PMC13645453; doi:10.1111/nhs.70431)
Supplement: Supplementary file 3 [file NHS-28-e70431-s003.docx]

**Supplementary Box S1. Operational criteria for nursing and midwifery relevance**

**General rule**

Nursing and midwifery relevance were operationalised through four non-mutually exclusive pathways: intervention-role relevance, end-user relevance, data-and-question relevance, and educational relevance. Studies were required to satisfy at least one pathway, based on evidence reported in the full text. Satisfaction of a relevance pathway did not override the separate eligibility requirements for a substantive ML role, an explicit causal estimand, and a corresponding identification strategy. Midwifery and midwifery education were included within the scope of the review alongside nursing. The operational criteria, qualifying evidence or applications, and conditions that were insufficient on their own are summarised below.

| **Pathway** | **Operational criterion** | **Qualifying evidence or applications** | **Not sufficient on its own** |
| --- | --- | --- | --- |
| **Intervention-role relevance** | Nurses or midwives had a substantive role in the intervention or service-delivery model being evaluated. An intervention was classified as nurse-led or midwife-led when nurses or midwives held primary responsibility and accountability for planning, clinical or organisational decision-making, coordination, or delivery, with discernible autonomy relative to medically led care (McParland et al., 2022; Ruksakulpiwat et al., 2025; Wong & Chung, 2006). Authors’ use of the term *nurse-led* or *midwife-led* alone was insufficient because these labels are not applied consistently across the literature (Terry et al., 2024). | Nurse-implemented or midwife-implemented interventions included substantive delivery or execution of a patient-care or service-delivery intervention within actual practice or workflow, such as an ML-triggered assessment, monitoring action, escalation process, care response, or follow-up intervention. | Use of the label *nurse-led* or *midwife-led* without supporting evidence; incidental participation; execution of medically directed care or peripheral tasks without a substantive role in the intervention. |
| **End-user relevance** | An ML-enabled tool or system was directed to nurses or midwives as intended end-users. A tool was classified as nurse-facing or midwife-facing when its output was delivered to nurses or midwives who were expected to interpret or act on it within an actual clinical or operational workflow (Mebrahtu et al., 2021; Randell et al., 2007). | Outputs could include alerts, predictions, risk scores, dashboards, recommendations, or decision-support interfaces. Relevant workflows included triage, monitoring, escalation of care, documentation, prioritisation, care coordination, communication, or clinical decision support. Registered nurses in advanced practice roles qualified when their role in receiving or acting on the output was documented. | Merely mentioning nurses or midwives among possible users; an unspecified nursing role; a tool directed jointly to several professional groups without a substantive and documented nursing or midwifery role. Nurse-facing or midwife-facing use was not automatically classified as nurse-led or midwife-led. |
| **Data-and-question relevance** | **Both conditions were required:** (1) nursing or midwifery data constituted a primary analytic source and (2) the causal question directly concerned nursing or midwifery care, assessment, monitoring, documentation, care delivery, or decision-making. Nursing documentation represents a substantive source of recorded nursing information (Bjerkan et al., 2021). | Eligible sources included nursing documentation or notes, midwifery records, bedside monitoring records, nursing assessments, care plans, structured nursing observations, or routinely recorded nursing-care data. | Nursing data alone when the causal question was primarily medical, administrative, or technical; isolated nursing variables without a nursing-related causal question; general clinical data collected in a setting where nurses happened to work. |
| **Educational relevance** | An ML-enabled application was used within nursing or midwifery education or training, and the causal question concerned a nursing- or midwifery-specific educational effect. The emphasis on competency- and outcome-based education was informed by the World Health Organization framework for health-worker education (World Health Organization, 2022). | Applications could include teaching, assessment, feedback, simulation, clinical-skills development, learner support, adaptive learning, or decision-support training. Outcomes could include discipline-specific knowledge, competency, clinical reasoning, clinical skills, simulation performance, care-planning ability, or preparation for professional practice. Nursing or midwifery learners had to constitute the target population; in multiprofessional samples, their results had to be separately identifiable. | Participation of nurses, midwives, or students alone; generic AI literacy, research skills, academic writing, technology acceptance, or broad professional-development outcomes without nursing- or midwifery-specific educational content and outcomes. |
| ***Note.*** The cited literature informed specific elements of the operational definitions; the four pathways and eligibility thresholds were developed for this review. **Abbreviations:** AI, artificial intelligence; ML, machine learning. | | | |

**References**

Bjerkan, J., Valderaune, V., & Olsen, R. M. (2021). Patient Safety Through Nursing Documentation: Barriers Identified by Healthcare Professionals and Students. *Frontiers in Computer Science*, *3*. https://doi.org/10.3389/fcomp.2021.624555

McParland, C., Johnston, B., & Cooper, M. (2022). A mixed‐methods systematic review of nurse‐led interventions for people with multimorbidity. *Journal of Advanced Nursing*, *78*(12), 3930–3951. https://doi.org/10.1111/jan.15427

Mebrahtu, T. F., Skyrme, S., Randell, R., Keenan, A.-M., Bloor, K., Yang, H., Andre, D., Ledward, A., King, H., & Thompson, C. (2021). Effects of computerised clinical decision support systems (CDSS) on nursing and allied health professional performance and patient outcomes: A systematic review of experimental and observational studies. *BMJ Open*, *11*(12), e053886. https://doi.org/10.1136/bmjopen-2021-053886

Randell, R., Mitchell, N., Dowding, D., Cullum, N., & Thompson, C. (2007). Effects of computerized decision support systems on nursing performance and patient outcomes: a systematic review. *Journal of Health Services Research & Policy*, *12*(4), 242–251. https://doi.org/10.1258/135581907782101543

Ruksakulpiwat, S., Pongsuwun, K., Junphongsri, P., Preeprem, C., Nguantad, S., & Samart, B. (2025). Nurse‐Led Interventions to Improve Health, Adherence, and Functional Outcomes in Adults and Older Adults With Multimorbidity: A Systematic Review of Randomized and Quasiexperimental Studies. *Journal of Nursing Management*, *2025*(1). https://doi.org/10.1155/jonm/6252049

Terry, D., Hills, D., Bradley, C., & Govan, L. (2024). Nurse‐led clinics in primary health care: A scoping review of contemporary definitions, implementation enablers and barriers and their health impact. *Journal of Clinical Nursing*, *33*(5), 1724–1738. https://doi.org/10.1111/jocn.17003

Wong, F. K. Y., & Chung, L. C. Y. (2006). Establishing a definition for a nurse‐led clinic: structure, process, and outcome. *Journal of Advanced Nursing*, *53*(3), 358–369. https://doi.org/10.1111/j.1365-2648.2006.03730.x

World Health Organization. (2022). *Global Competency and Outcomes Framework for Universal Health Coverage*. https://www.who.int/publications/i/item/9789240034662
